# Psathyrellins A–E, Antibacterial Guanacastane Diterpenoids from Mushroom *Psathyrella candolleana*

**DOI:** 10.1007/s13659-021-00316-x

**Published:** 2021-07-06

**Authors:** Han Wu, Hui-Xiang Yang, Zheng-Hui Li, Tao Feng, Ji-Kai Liu

**Affiliations:** 1 Anhui Key Laboratory of Modern Chinese Materia Medica, School of Pharmacy, Anhui University of Chinese Medicine, Hefei, 230012 People’s Republic of China; 2 School of Pharmaceutical Sciences, South-Central University for Nationalities, Wuhan, 430074 People’s Republic of China

**Keywords:** *Psathyrella candolleana*, Guanacastane diterpenoids, Antibacterial activity

## Abstract

**Abstract:**

Five previously undescribed guanacastane diterpenoids, namely psathyrellins A–E (**1**–**5**), were obtained from cultures of the mushroom *Psathyrella candolleana*. Their structures with absolute configurations were elucidated by extensive spectroscopic methods. Compounds **1**–**3** showed antibacterial activity against four strains with MIC values in a range of 16–128 μg/mL.

**Graphic Abstract:**

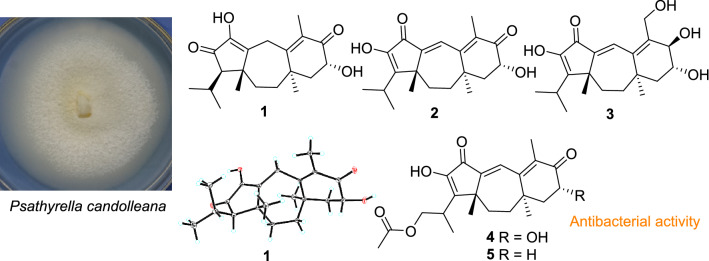

**Supplementary Information:**

The online version contains supplementary material available at 10.1007/s13659-021-00316-x.

## Introduction

**Fig. 1:**
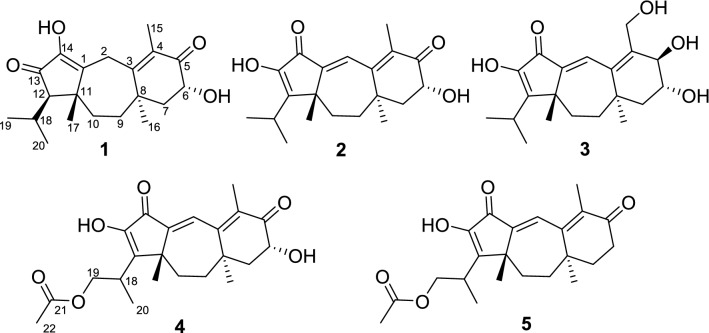
Structures of compounds **1**–**5**

The basidiomycete *Psathyrella candolleana* (Psathyrellaceae) is a small agaric usually found in the vicinity of recently dead hardwood trees. It has a wide distribution on lawns or pastures in Europe and North America. Previous pharmacological studies indicated that the extracts of *P*. *candolleana* showed a marked anticlastogenic effect against DNA damage [1‒3], while chemical investigations on this fungus have demonstrated guanacastane-type diterpenoids [4, 5], which possess a unique 5/7/6 tricyclic backbone that have been found only in several fungal species. These diterpenoids have been detected to possess antibacterial and cytotoxic properties [5‒7]. As our long-term chemical studies on higher fungi [8–13], a chemical investigation on this mushroom resulted in the isolation of five new guanacastane diterpenoids, namely psathyrellins A–E (**1**–**5**, Fig. 1). Their structures were elucidated by extensive spectroscopic methods, while their absolute configurations were established by the single crystal X-ray diffraction and CD spectra. All compounds were evaluated for their antibacterial activity against four strains. Herein, the isolation, structural elucidation, and the biological activities of these isolates are reported.

## Results and Discussion

**Fig. 2:**
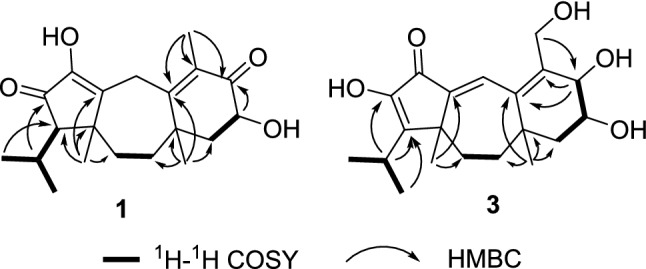
Key ^1^H–^1^H COSY and HMBC correlations of **1** and **3**

**Fig. 3:**
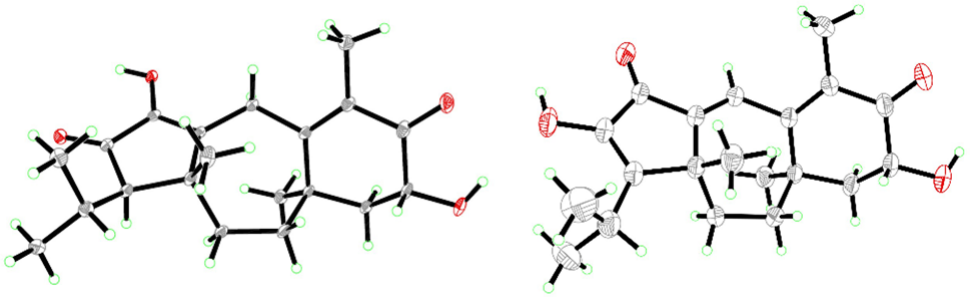
ORTEP diagrams of **1** (left) and **2** (right)

Psathyrellin A (**1**) was isolated as colorless crystals. Its molecular formula was determined as C_20_H_28_O_4_ on the basis of HRESIMS at *m/z* 333.20584 (calcd for C_20_H_28_O_4_ [M + H]^+^, 333.30604), implying seven degrees of unsaturation. The IR absorption bands at 3421, 1710, 1640, 1462 cm^−1^ revealed the existence of hydroxy, carbonyl, and double bonds. In the ^1^H NMR spectrum (Table 1), three singlets at *δ*_H_ 1.06, 1.09, 1.85, two doublets at *δ*_H_ 0.79 (3H, d, *J* = 6.9 Hz), 1.12 (3H, d, *J* = 6.9 Hz) were readily assigned for five methyl groups. In addition, one doublet at *δ*_H_ 4.46 (1H, dd, *J* = 14.0, 5.2 Hz) indicated an oxidized methine carbon. In the ^13^C NMR spectrum, a total of 20 carbon resonances were detected. They were classified into five CH_3_, four CH_2_, three CH, and eight non-protonated carbons by the DEPT and HSQC spectra (Table 2). Of them, two carbonyl signals at *δ*_C_ 200.8 (s, C-5) and 205.4 (s, C-13), and four olefinic signals four two double bonds at *δ*_C_ 150.2 (s, C-1), 162.5 (s, C-3), 130.5 (s, C-4), and 149.3 (s, C-14) occupied four degrees of unsaturation, suggesting a tricyclic backbone of **1**. All these data, as well as the literature survey [5, 6, 14], suggested that **1** should be a guanacastane diterpenoid characterized with a 5/7/6-fused ring system. Preliminary analysis of 2D NMR data (especially the HMBC data) revealed two *α*,*β*-unsaturated keto moieties in rings A and B, respectively (Fig. 2). A HMBC correlation from *δ*_H_ 4.46 (1H, dd, *J* = 14.0, 5.2 Hz, H-6) to C-5, as well as a ^1^H–^1^H COSY correlation between H-6 and H-7, suggested a hydroxy group placed at C-6. After many attempts, a single crystal of **1** was obtained from methanol, while the single crystal X-ray diffraction revealed the absolute configuration of **1** as shown in Fig. 3 (Flack parameter = 0.05(3); CCDC: 2068966).

**Table 1:** ^1^H NMR Data for **1**–**5** in Methanosl-*d*_4_ (*δ* in ppm, *J* in Hz)

No.	**1**	**2**	**3**	**4**	**5**
2	3.63, m 3.46, d (0.9)	6.99, br s	7.07, d (2.2)	7.03, q (1.2)	7.05, d (1.3)
5			4.12, d (7.9)		
6	4.46, dd (14.0, 5.2)	4.55, dd (14.0, 5.4)	3.86, ddd (12.0, 7.9, 3.7)	4.56, dd (14.0, 5.4)	2.74, m; 2.41, m
7	1.99, m 1.82, d (6.6)	2.11, dd (12.9, 5.4) 1.96, m	1.74, dd (13.0, 3.8) 1.65, t (12.7)	2.11, dd (12.9, 5.5) 1.95, m	2.06, dd (13.5, 4.4) 1.92, m
9	2.26, td (14.5, 13.9, 3.9) 1.57, m	2.58, td (14.3, 3.1) 1.72, m	2.27, ddd (13.2, 12.8, 3.1) 1.57, m	2.58, m 1.72, m	1.59, ddd (14.4, 4.7, 3.2) 2.45, m
10	1.79, m 1.54, m	1.98, d (3.5) 1.70, q (3.7)	1.83, dt (13.9, 3.5) 1.51, dd (13.9, 3.1)	1.90, m 1.78, dd (14.1, 3.4)	1.89, m 1.76, dd (13.7, 3.1)
12	1.96, d (2.8)				
15	1.85, s	1.74, s	4.19, m	1.75, s	1.71, s
16	1.09, s	1.09, s	0.97, d (11.1)	1.10, s	1.08, s
17	1.06, s	1.11, s	1.09, d (7.2)	1.11, s	1.11, s
18	2.05, m	2.51, m	2.5, m	2.72, m	2.70, m
19	0.79, d (6.9)	1.29, dd (10.4, 6.9)	1.29, d (6.9)	4.40, dd (10.5, 8.1) 4.30, dd (10.5, 7.1)	4.40, dd (10.5, 8.1) 4.30, dd (10.5, 7.2)
20	1.12, d (6.9)	1.29, dd (10.4,6.9)	1.27, d (6.9)	1.27, d (6.9)	1.27, d (7.0)
22				1.99, s	1.99, s

**Table 2:** ^13^C NMR Data for **1**–**5** in Methanol-*d*_4_ (150 MHz, *δ* in ppm)

No.	**1**	**2**	**3**	**4**	**5**
1	150.2, C	142.8, C	143.0, C	142.7, C	142.9, C
2	30.9, CH_2_	129.0, CH	129.7, CH	129.1, CH	129.2, CH
3	162.5, C	158.6, C	152.1, C	158.5, C	159.2, C
4	130.5, C	131.5, C	138.5, C	131.6, C	133.6, C
5	200.8, C	200.8, C	75.0, CH	200.8, C	200.3, C
6	69.8, CH	69.7, CH	71.0, CH	69.7, CH	34.5, CH_2_
7	50.2, CH	48.1, CH_2_	47.2, CH_2_	48.1, CH_2_	39.3, CH_2_
8	41.8, C	39.2, C	39.1, C	39.2, C	38.1, C
9	36.4, CH_2_	35.7, CH_2_	38.5, CH_2_	35.7, CH_2_	35.7, CH_2_
10	42.5, CH_2_	31.5, CH_2_	31.1, CH_2_	31.6, CH_2_	31.3, CH_2_
11	45.3, C	45.1, C	45.1, C	44.9, C	44.9, C
12	62.9, CH	158.6, C	158.5, C	152.7, C	152.7, C
13	205.4, C	152.2, C	139.5, C	152.8, C	152.7, C
14	149.3, C	190.1, C	190.8, C	189.9, C	190.1, C
15	11.8, CH_3_	12.3, CH_3_	59.4, CH_2_	12.3, CH_3_	12.3, CH_3_
16	25.2, CH_3_	27.3, CH_3_	26.7, CH_3_	27.3, CH_3_	26.2, CH_3_
17	17.1, CH_3_	20.5, CH_3_	20.4, CH_3_	20.7, CH_3_	21.0, CH_3_
18	28.9, CH	27.3, CH	27.3, CH	32.3, CH	32.3, CH
19	19.3, CH_3_	20.6, CH_3_	20.8, CH_3_	67.1, CH_2_	67.1, CH
20	23.6, CH_3_	20.7, CH_3_	20.7, CH_3_	15.5, CH_3_	15.5, CH_3_
21				172.8, C	172.7, C
22				20.8, CH_3_	20.8, CH_3_

Psathyrellin B (**2**) was isolated as colorless crystals. Its molecular formula was determined as C_20_H_26_O_4_ by HRESIMS data at *m/z* 331.19031 [M + H]^+^ (calcd for C_20_H_27_O_4_^+^, 331.19039). The UV data at 282 nm suggested a conjugated system in **2**. The 1D and 2D NMR spectra revealed similar patterns to those of **1** except that one more double bond in **2**. In the HMBC spectrum, correlations from *δ*_H_ 1.11 (3H, s, H_3_-17) to *δ*_C_ 142.8 (s, C-1) and 158.6 (s, C-12), from *δ*_H_ 6.99 (1H, br s, H-2) to C-1 and *δ*_C_ 158.6 (s, C-3), and from *δ*_H_ 1.74 (3H, s, H_3_-15) to C-3 and *δ*_C_ 131.5 (s, C-4) suggested that three double bonds were distributed at C-12/C-13, C-1/C-2, and C-3/C-4, respectively. Detailed analysis of 2D NMR data suggested that the other parts of **2** were the same to those of **1**. A single crystal X-ray diffraction not only proved the planar structure, but also determined the absolute configuration of **1** as shown in Fig. 3 (Flack parameter = − 0.12(14); CCDC: 2068967).

**Fig. 4:**
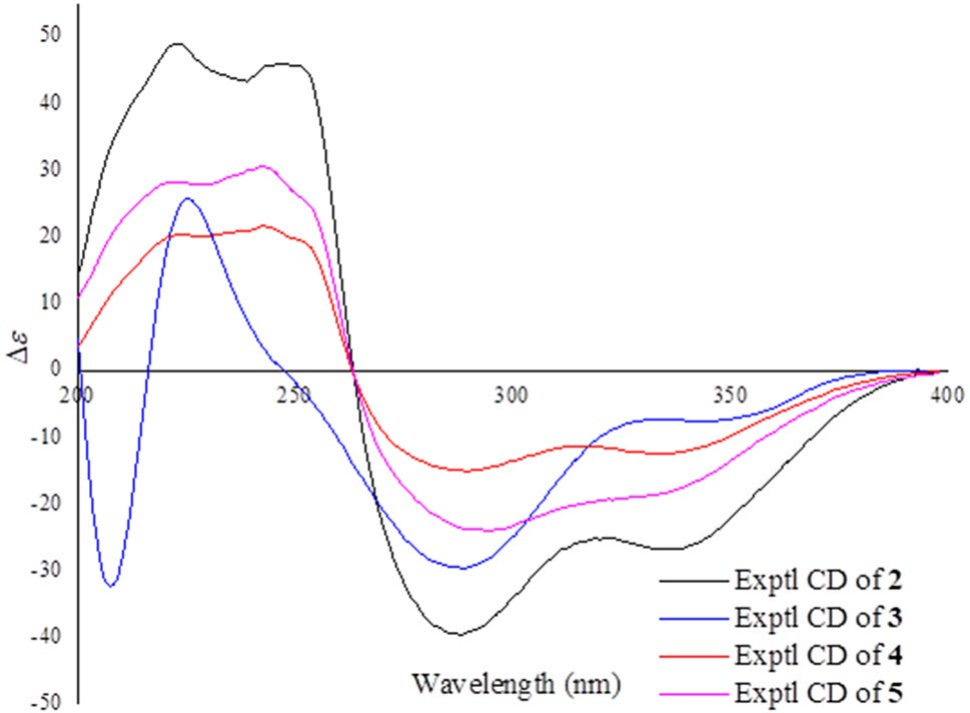
CD curves of compounds **2**–**5**

Psathyrellin C (**3**) was isolated as a yellow oil. The molecular formula was determined as C_20_H_28_O_5_ on the basis of HRESIMS data at *m/z* 349.20093 [M + H]^+^ (calcd for C_20_H_29_O_5_^+^, 349.20095). All NMR data suggested that **3** was structurally similar to that of **2** (Fig. 2). In compound **3**, C-15 was oxidized into a hydroxymethylene at *δ*_C_ 59.4. In addition, C-5 was reduced into a hydroxymethine at *δ*_C_ 75.0. These information were supported by the HMBC and ^1^H–^1^H COSY data. In the ROESY spectrum, correlation of H-6 with H-9 suggested that OH at C-6 should be *α* oriented. Based on this information, the coupling constant of *J*_5,6_ = 7.9 Hz indicated that the OH at C-5 should be *β* oriented. The CD spectrum of **3** revealed similar Cotton effects with those of **2** (Fig. 4), indicating the absolute configuration of **3** to be the same to that of **2**.

Psathyrellin D (**4**) was isolated as a yellow oil. The molecular formula was determined as C_22_H_28_O_6_ on the basis of HRESIMS data at *m/z* 411.17749 [M + Na]^+^ (calcd for C_22_H_28_O_6_Na^+^, 411.17781). All spectroscopic data of **4** were similar to those of **2** excepted two additional carbons at *δ*_C_ 20.8 (q) and 172.8 (s) in **4** that were easily assigned as an *O*-acetyl moiety. The HMBC correlations from *δ*_H_ 4.40 and 4.30 to *δ*_C_ 172.8 (s) and from *δ*_H_ 1.27 (3H, d, *J* = 6.9 Hz, H_3_-20) to *δ*_C_ 32.3 (d, C-18), 67.1 (t, C-19) suggested that the *O*-acetyl moiety should be placed at C-19. Detailed analysis of 2D NMR data suggested that the other parts of **4** were the same to those of **2**. The CD spectrum almost showed the same curve to that of **2**, indicating the absolute configuration of the main backbone in **4** to be the same to that of **2** (Fig. 4). However, the stereochemistry of C-18 could not be established currently.

Psathyrellin E (**5**) was isolated as a yellow oil. The molecular formula was determined as C_22_H_28_O_5_ on the basis of HRESIMS data at *m/z* 373.20084 [M + H]^+^ (calcd for C_22_H_29_O_5_^+^, 373.20095). According to analysis of 1D and 2D NMR data, compound **5** was easily identified as a 19-*O*-acetyl derivative of **2**, which was also similar to **4**. The only difference was that the hydroxymethine of C-6 in **4** was reduced into a methylene at *δ*_C_ 34.5 (t, C-6) in **5**, as supported by the HMBC correlation from H-6 to C-5 and the ^1^H–^1^H COSY data between H-6 and H-7. The CD spectrum almost indicated the same curve to that of **2**, indicating the absolute configuration of the backbone in **5** to be the same to that of **2** (Fig. 4). However, the stereochemistry of C-18 could not be established currently.

Compounds **1**–**5** were evaluated for their antibacterial activities against *Escherichia coli*, *Staphylococcus aureus*, *Salmonella enterica*, and *Pseudomonas aeruginosa*. As a result, compounds **1**–**3** showed antibacterial activity with MIC values in a range of 16–128 μg/mL (Table 3).

**Table 3:** Antibacterial activity of **1**–**5** (MIC, μg/mL)

Comp	*Escherichia coli*	*Staphylococcus aureus* subsp. *aureus*	*Salmonella enterica* subsp. *enterica*	*Pseudomonas aeruginosa*
**1**	16	64	64	16
**2**	16	16	32	16
**3**	64	128	128	64
**4**	> 128	> 128	> 128	> 128
**5**	> 128	> 128	> 128	> 128
Chloramphenicol	4	4	4	1

## Experimental Section

### General Experimental Procedures

Optical rotations were measured on a Rudolph Autopol IV polarimeter. UV spectra were obtained on a UH5300 UV–VIS Double Beam Spectrophotometer. IR spectra were obtained by using a Shimadzu Fourier Transform Infrared spectrometer with KBr pellets. NMR spectra were acquired with a Bruker Avance III 600 instrument. CD spectra were recorded with an Applied Photophysics Chirascan-Plus spectrometer. High resolution electrospray ionization mass spectra (HRESIMS) were recorded on a LC–MS system consisting of a Q Exactive™ Orbitrap mass spectrometer with an ESI ion source used in ultra-high resolution mode (140,000, at *m/z* 200) and a Dionex UltiMate 3000 RSLC UPLC system. Silica gel (200–300 mesh and 500–800 mesh), RP-18 gel (40–75 µm) and Sephadex LH-20 were used for column chromatography (CC). Preparative HPLC was performed on an Agilent 1260 liquid chromatography system with a Zorbax SB-C18 (5 µm, 9.4 × 150 mm) column, a Daicel chiral column (AS-H, 5 µm, 4.6 × 250 mm) and a DAD detector.

### Fungal Material and Cultivation Conditions

Fruiting bodies of *P*. *candolleana* were collected at Jingdong of Yunnan Province, China in 2003. They were identified by Prof. Zhu-Liang Yang of Kunming Institute of Botany, Chinese Academy of Sciences. The voucher specimen (NO.CGBWSHF00118.2) was deposited at School of Pharmaceutical Sciences, South-Central University for Nationalities. The strains were cultured in PDA and stored at − 4 °C. Culture medium was composed of glucose (5%), pork pepton (0.15%), yeast (0.5%), KH_2_PO_4_ (0.05%) and MgSO_4_ (0.05%). Initial pH was adjusted to 6.0, the fermentation was first carried out on an erlenmeyer flask for 6 days till the mycelium biomass reached to the maximum. Then it was transferred to rice medium at 24 °C in dark culture for 40 days. The rice medium in each 250 mL-Erlenmeyer flask was composed of rice (50 g) and water (50 mL). A total of 180 bottles were used in this study.

### Extraction and Isolation

The rice fermentation (9 kg) was extracted four times with EtOAc. The organic layer was evaporated to give a crude extract (90 g). The extract was subjected to silica gel CC (200–300 mesh) eluted with a gradient solvent system of petroleum ether (PE)/Me_2_CO (from 20:1 to 1:1) to afford eight fractions A–H. Fraction C (3.8 g) was isolated by CC over silica gel using PE/Me_2_CO (6/1) to give subfractions C1–C6. Compound **1** was deposited from fraction C4 as colorless crystals (6 mg; purity > 95%). Fraction E (4 g) was first isolated by silica gel CC (200–300 mesh) eluted with PE/Me_2_CO (5/1) to give five subfractions E1-E5. Fraction E2 (800 mg) was further isolated by CC using RP-C18 silica gel (MeOH/H_2_O from 6/4 to 9/1) to give subfractions E2a-E2e. HPLC preparation (MeCN/H_2_O from 7/3 to 8/2 in 20 min) on fraction E2d (82 mg) afforded compounds **3** (1.8 mg, retention time (t_*R*_) = 12.1 min; purity 90%), **4** (2.8 mg, t_*R*_ = 12.8 min; purity 90%), and **2** (4.3 mg, t_*R*_ = 14.6 min; purity > 95%). Fraction E2e (70 mg) was separated by CC over Sephadex LH-20 (MeOH) to give a mixture. The mixture was subjected to HPLC (MeCN/H_2_O from 7/3 to 8/2 in 20 min) to give compound **5** (2.6 mg, t_*R*_ = 15.1 min; purity 90%).

### Spectroscopic Data of Compounds

#### Psathyrellin A (1)

Colorless crystals (MeOH); [*α*]_D_^15^ – 208.1 (*c* 0.22, MeOH); UV (MeOH) *λ*_max_ (log *ε*) 192 (3.36), 244 (3.18) nm; IR (KBr) *ν*_max_ 3421, 3349, 2829, 1710, 1702, 1640, 1462, 1038 cm^−1^; ^1^H (600 MHz) and ^13^C NMR (150 MHz) data (methanol-*d*_4_), see Table 1; HRESIMS *m/z* 333.20584 [M + H]^+^ (calcd for C_20_H_29_O_4_^+^, 333.20604).

#### Psathyrellin B (2)

Colorless crystals (MeOH); [*α*]_D_^15^ – 239.2 (*c* 0.18, MeOH); UV (H_2_O) *λ*_max_ (log *ε*) 190 (3.34), 252 (3.20), 282 (2.86) nm; ECD (MeOH) *λ*_max_ (Δ*ε*) 223 (+49), 251 (+45), 287 (–39), 339 (–27) nm; IR (KBr) *ν*_max_ 3432, 3346, 2910, 1711, 1706, 1642, 1446, 1036 cm^−1^; ^1^H (600 MHz) and ^13^C NMR (150 MHz) data (methanol-*d*_4_), see Table 1; HRESIMS *m/z* 331.19031 [M + H]^+^ (calcd for C_20_H_27_O_4_^+^, 331.19039).

#### Psathyrellin C (3)

Yellow oil; [*α*]_D_^15^ – 182.9 (*c* 0.12, MeOH); ECD (MeOH) *λ*_max_ (Δ*ε*) 209 (–33), 224 (+26), 287 (–29), 341 (–8) nm; ^1^H (600 MHz) and ^13^C NMR (150 MHz) data (methanol-*d*_4_), see Table 1; HRESIMS *m/z* 349.20093 [M + H]^+^ (calcd for C_20_H_29_O_5_^+^, 349.20095).

#### Psathyrellin D (4)

Yellow oil; [*α*]_D_^15^ – 221.9 (*c* 0.14, MeOH); ECD (MeOH) *λ*_max_ (Δ*ε*) 222 (+20), 243 (+22), 287 (–15), 338 (–12) nm; ^1^H (600 MHz) and ^13^C NMR (150 MHz) data (methanol-*d*_4_), see Table 1; HRESIMS *m/z* 411.17749 [M + Na]^+^ (calcd for C_22_H_28_O_6_Na^+^, 411.17781).

#### Psathyrellin E (5)

Yellow oil; [*α*]_D_^15^ – 292.4 (*c* 0.12, MeOH); ECD (MeOH) *λ*_max_ (Δ*ε*) 221 (+27), 242 (+31), 290 (–24), 338 (–18) nm; ^1^H (600 MHz) and ^13^C NMR (150 MHz) data (methanol-*d*_4_), see Table 1; HRESIMS *m/z* 373.20084 [M + H]^+^ (calcd for C_22_H_29_O_5_^+^, 373.20095).

#### X-Ray Crystallographic Data for Psathyrellin A (1)

C_20_H_28_O_4_, *M* = 332.42, *a* = 14.0862(3) Å, *b* = 7.26370(10) Å, *c* = 17.3636(3) Å, *α* = 90°, *β* = 99.5460(10)°, *γ* = 90°, *V* = 1752.01(5) Å^3^, *T* = 100.(2) K, space group *P*1211, *Z* = 4, *μ*(Cu Kα) = 0.692 mm^−1^, 33652 reflections measured, 6741 independent reflections (*R*_*int*_ = 0.0259). The final *R*_*1*_ values were 0.0306 [*I* > 2*σ*(*I*)]. The final *wR*(*F*^2^) values were 0.0799 [*I* > 2*σ*(*I*)]. The final *R*_*1*_ values were 0.0307 (all data). The final *wR*(*F*^2^) values were 0.0800 (all data). The goodness of fit on *F*^2^ was 1.056. Flack parameter = 0.05(3). CCDC: 2068966 (www.ccdc.cam.ac.uk).

#### X-Ray Crystallographic Data for Psathyrellin B (2)

C_20_H_26_O_4_, *M* = 330.41, *a* = 8.0026(8) Å, *b* = 8.3729(9) Å, *c* = 14.4078(15) Å, *α* = 90.00°, *β* = 105.783(3)°, *γ* = 90.00°, *V* = 929.00(17) Å^3^, T = 150.(2) K, space group *P*1211, *Z* = 2, *μ*(CuKα) = 1.54178 mm^−1^, 12059 reflections measured, 3552 independent reflections (*R*_*int*_ = 0.0456). The final *R*_*1*_ values were 0.0559 [*I* > 2*σ*(*I*)]. The final *R*_*1*_ values were 0.0415 (all data). The final *wR*(*F*^2^) values were 0.1156 (all data). The goodness of fit on *F*^2^ was 1.092. Flack parameter = − 0.12(14). CCDC: 2068967 (www.ccdc.cam.ac.uk).

### Antibacterial Assay

The tested bacteria strains *Escherichia coli* ATCC25922, *Staphylococcus aureus* subsp. *aureus* ATCC29213, *Salmonella enterica* subsp. *enterica* ATCC14028, *Pseudomonas aeruginosa* ATCC27853 were purchased from China General Microbiological Culture Collection Center, (CGMCC). All these strains were cultured in Mueller Hinton broth (MHB) (Guangdong Huankai Microbial Sci. &Tech. Co., Ltd.) at 37 °C overnight with shaking (200 rpm). A sample of each culture was then diluted 40-fold in fresh MHB broth and incubated with shaking (200 rpm) at 37 °C for 2.5 h. The resultant mid-log phase cultures were diluted to a concentration of 5 × 10^5^ CFU/mL, then 50 mL was added to each well of the compound-containing plates. The minimum inhibition concentration (MIC) was determined by measuring bacterial growth after 24 h on performing 1:2 serial dilutions of each compound ranging from 1 to 128 μg/mL. Chloramphenicol was used as a positive control.

## Supplementary Information

Below is the link to the electronic supplementary material.

Supplementary file1 (PDF 2820 KB)

Supplementary file2 (CIF 1031 KB)

Supplementary file3 (CIF 379 KB)
