# Supplementary material for: Psathyrellins A–E, Antibacterial Guanacastane Diterpenoids from Mushroom Psathyrella candolleana
Source: Nat Prod Bioprospect. 2021 Jul 6;11(4):447–52. doi: 10.1007/s13659-021-00316-x (PMC8275727; doi:10.1007/s13659-021-00316-x)

Supporting Information for

Psathyrellins A–E, antibacterial guanacastane diterpenoids from mushroom *Psathyrella candolleana*

Han Wu[1,2], Hui-Xiang Yang[2], Zheng-Hui Li[2], Tao Feng[1,2*] and Ji-Kai Liu[1,2*]

[1]*School of Pharmacy, Anhui University of Chinese Medicine, Anhui Key Laboratory of Modern Chinese Materia Medica, Hefei 230012, People's Republic of China*

[2]*School of Pharmaceutical Sciences, South-Central University for Nationalities, Wuhan 430074, People's Republic of China*

[*]Corresponding authors: tfeng@mail.scuec.edu.cn (T. Feng); liujikai@mail.scuec.edu.cn (J. K. Liu)

Contents

Figure 1S–7S: NMR and MS spectra of psathyrellin A (**1**)…………………………………… p2S-5S

Figure 8S–15S: NMR, MS, and CD spectra of psathyrellin B (**2**)………………………… p6S-9S

Figure 16S–23S: NMR, MS, and CD spectra of psathyrellin C (**3**)………………………p10S-13S

Figure 24S–31S: NMR, MS, and CD spectra of psathyrellin D (**4**)………………………p14S-17S

Figure 32S–39S: NMR, MS, and CD spectra of psathyrellin E (**5**)………………………p18S-21S

Figure 1S. ^{1}H NMR (600 MHz, methanol-d_4) spectrum of psathyrellin A (**1**)

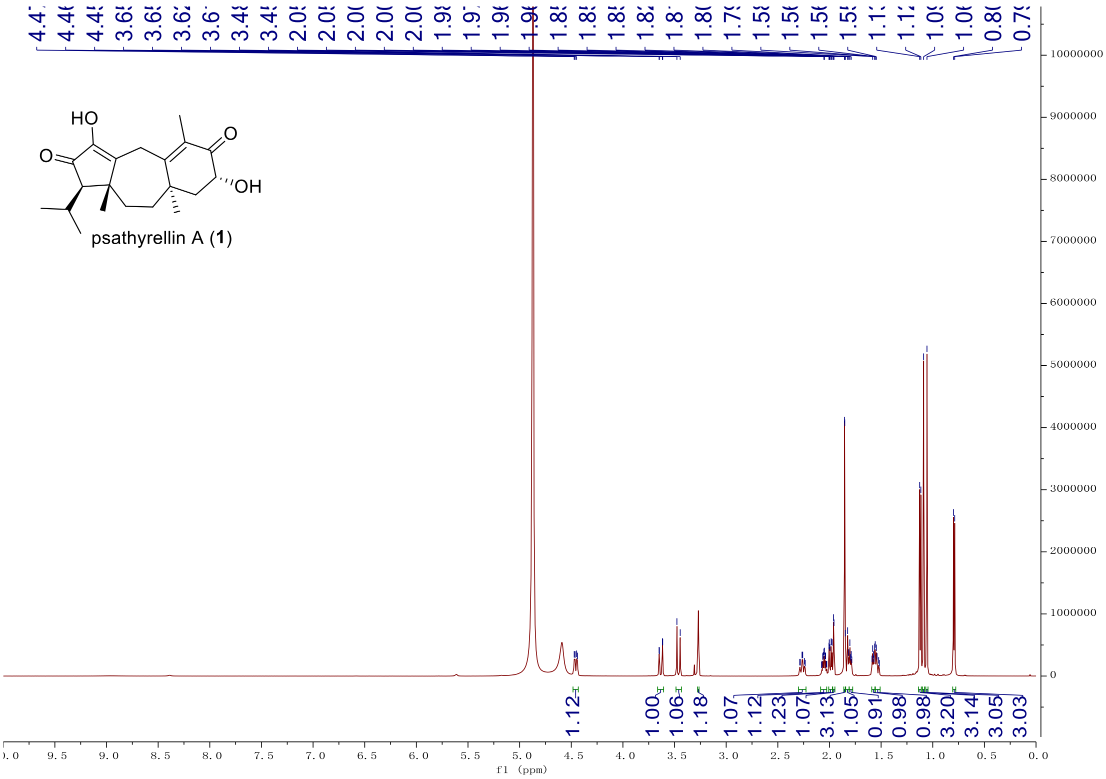

Figure 2S. ^{13}C NMR (150 MHz, methanol-d_4) spectrum of psathyrellin A (**1**)

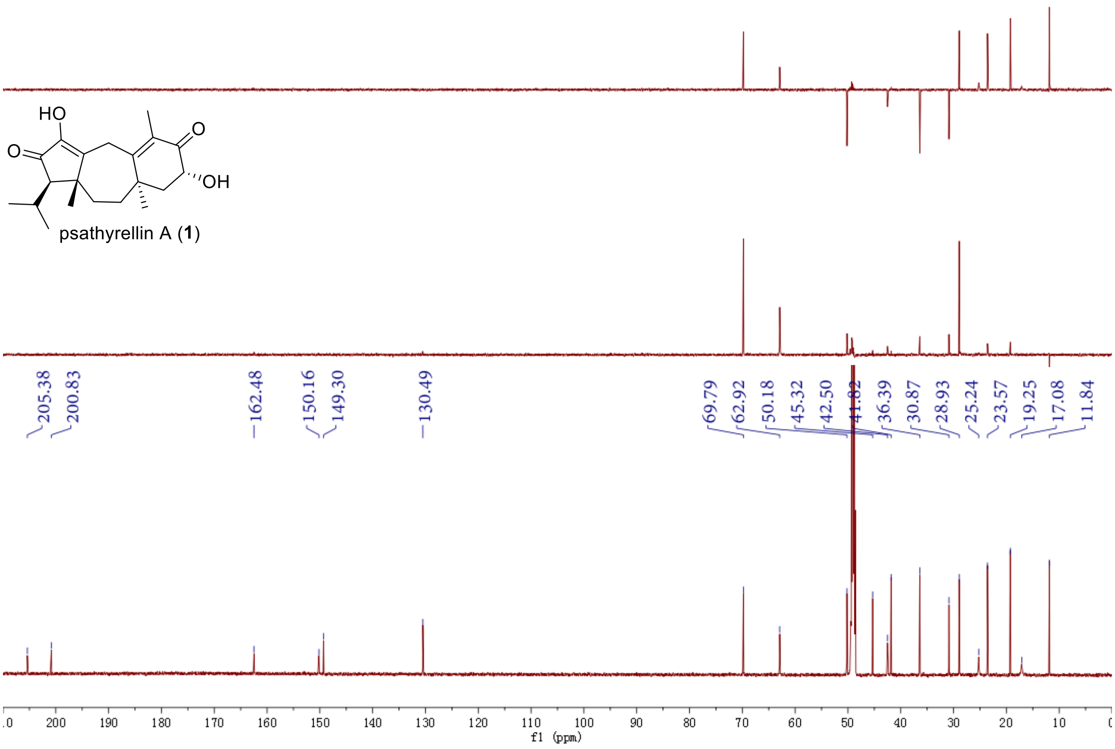

Figure 3S. ^{1}H-^{1}H COSY spectrum of psathyrellin A (**1**)

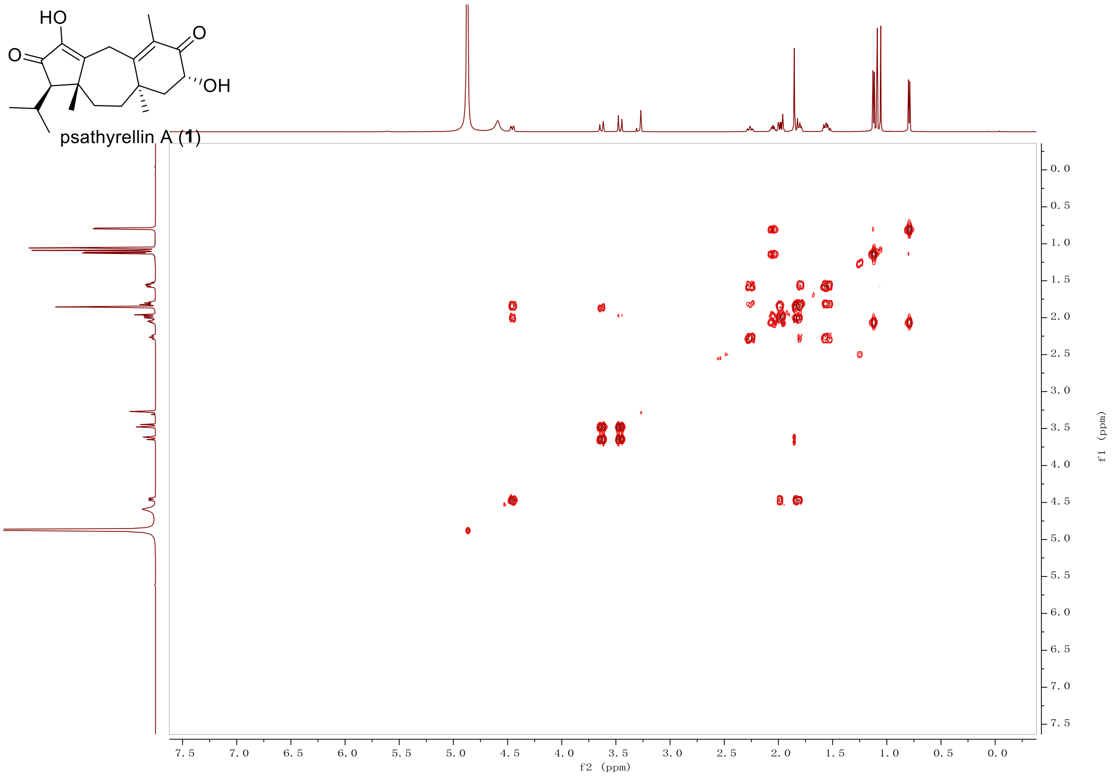

Figure 4S. HQSC spectrum of psathyrellin A (**1**)

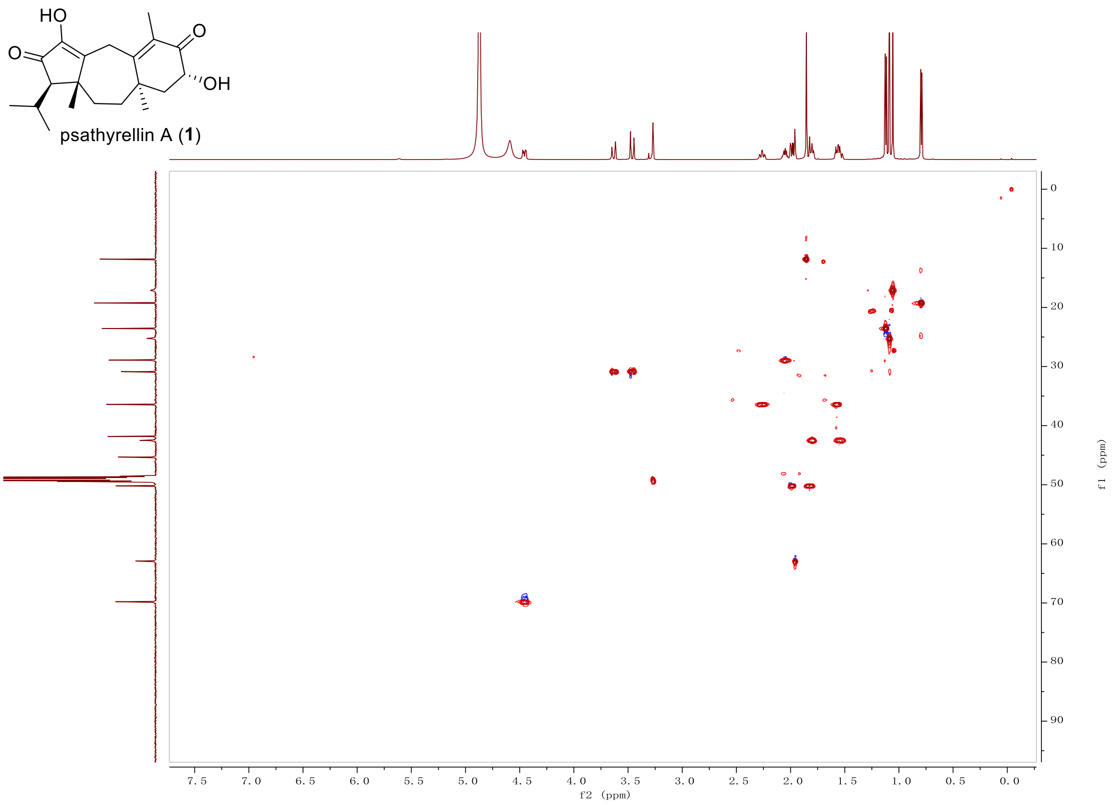

Figure 5S. HMBC spectrum of psathyrellin A (**1**)

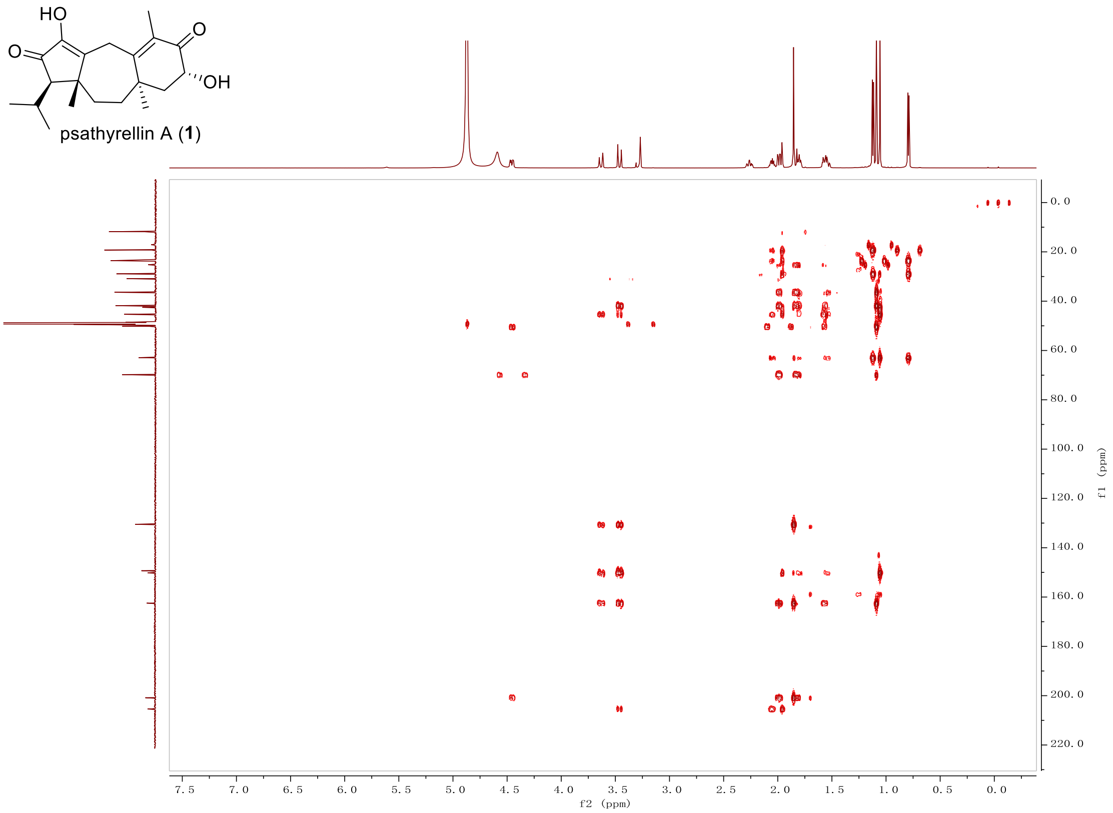

Figure 6S. ROESY spectrum of psathyrellin A (**1**)

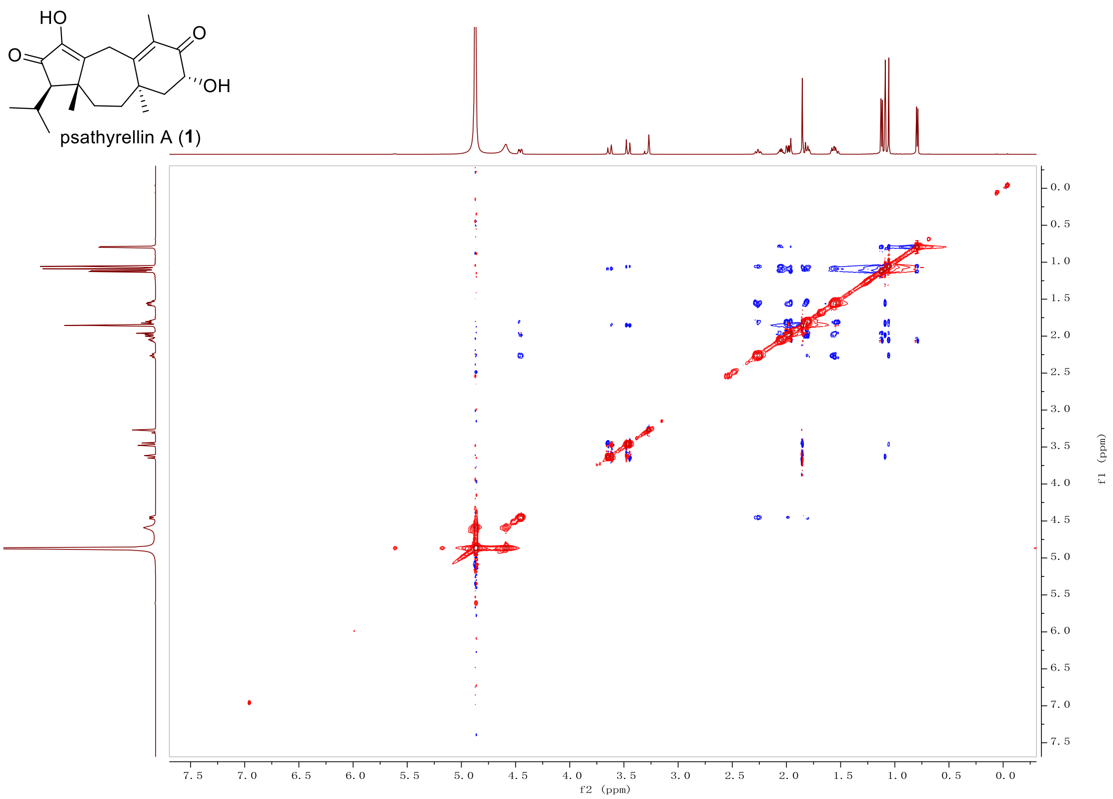

Figure 7S. HRESIMS of psathyrellin A (**1**)

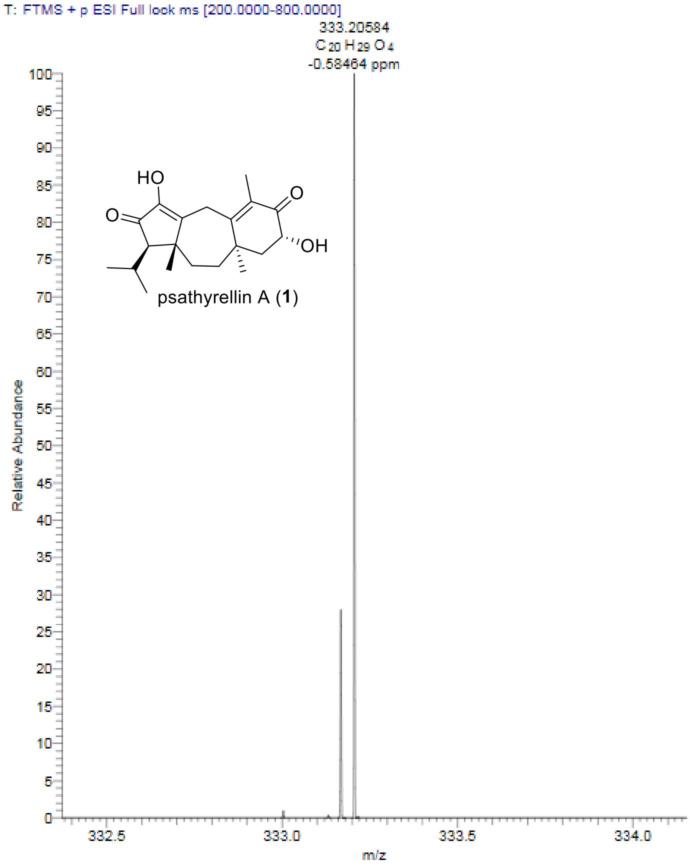

Figure 8S. ^{1}H NMR (600 MHz, methanol-d_4) spectrum of psathyrellin B (**2**)

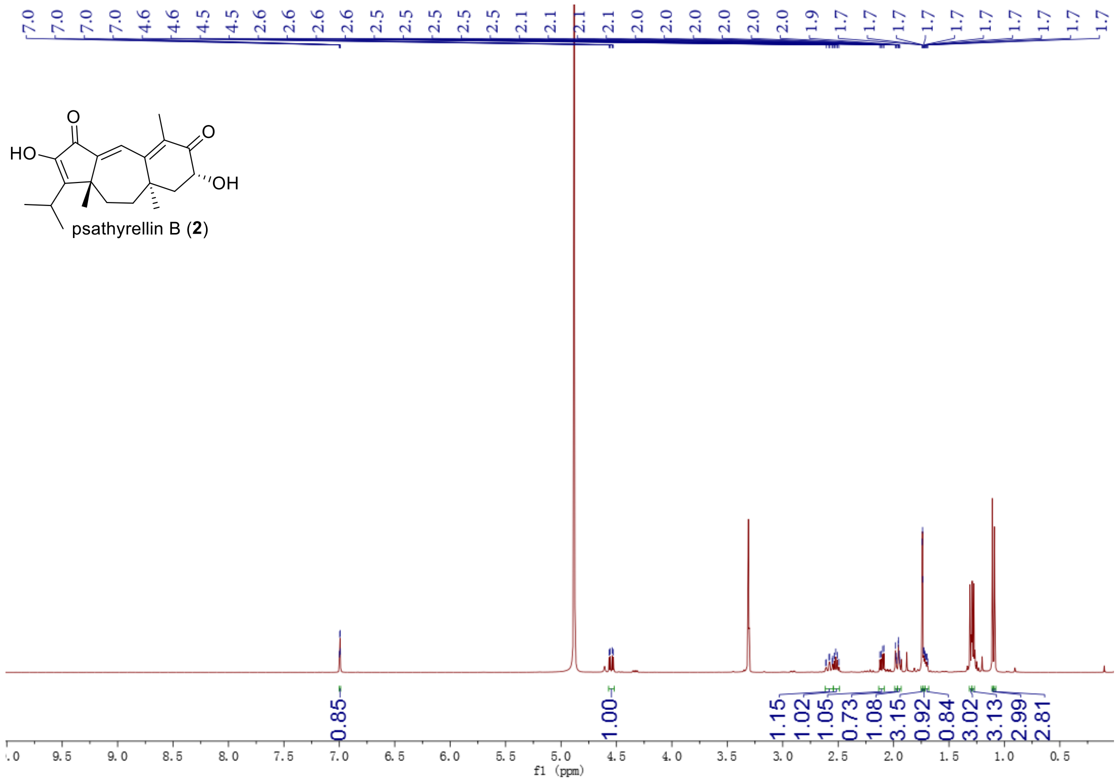

Figure 9S. ^{13}C NMR (150 MHz, methanol-d_4) spectrum of psathyrellin B (**2**)

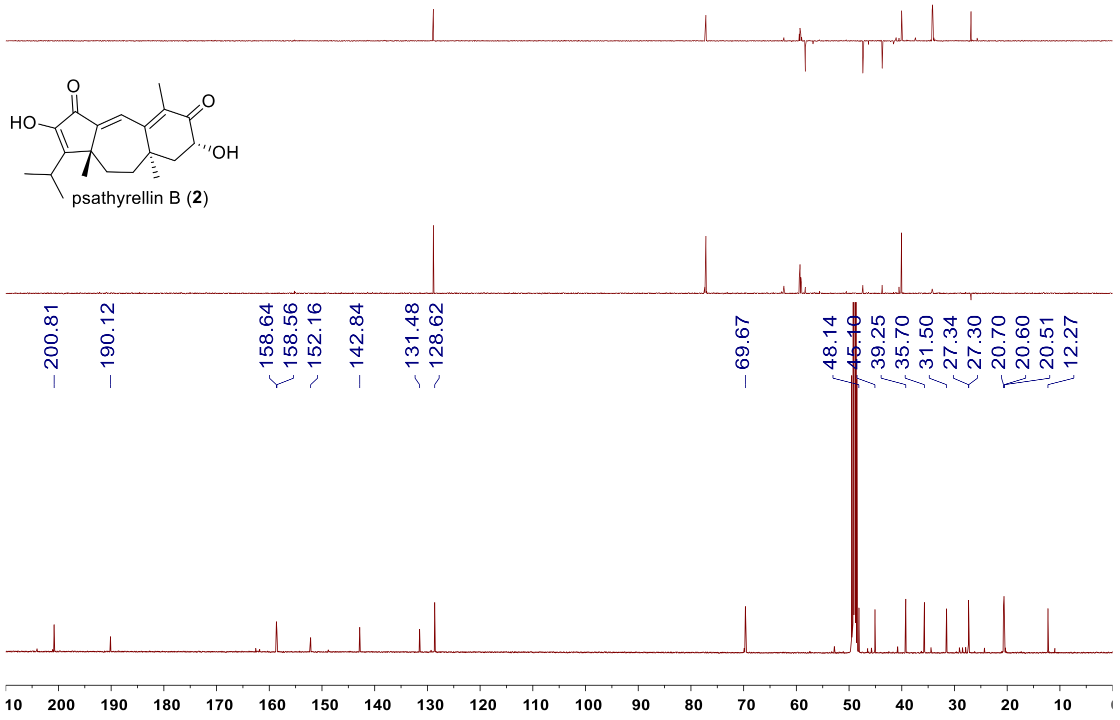

Figure 10S. ^{1}H-^{1}H COSY spectrum of psathyrellin B (**2**)

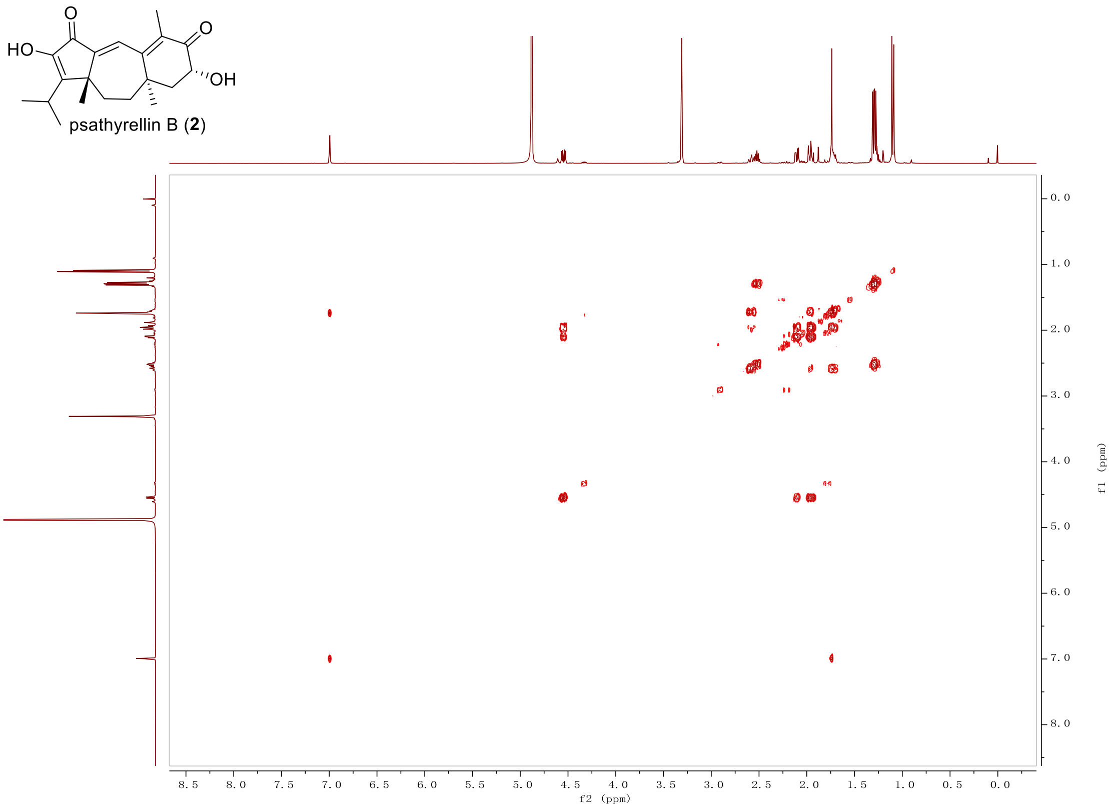

Figure 11S. HQSC spectrum of psathyrellin B (**2**)

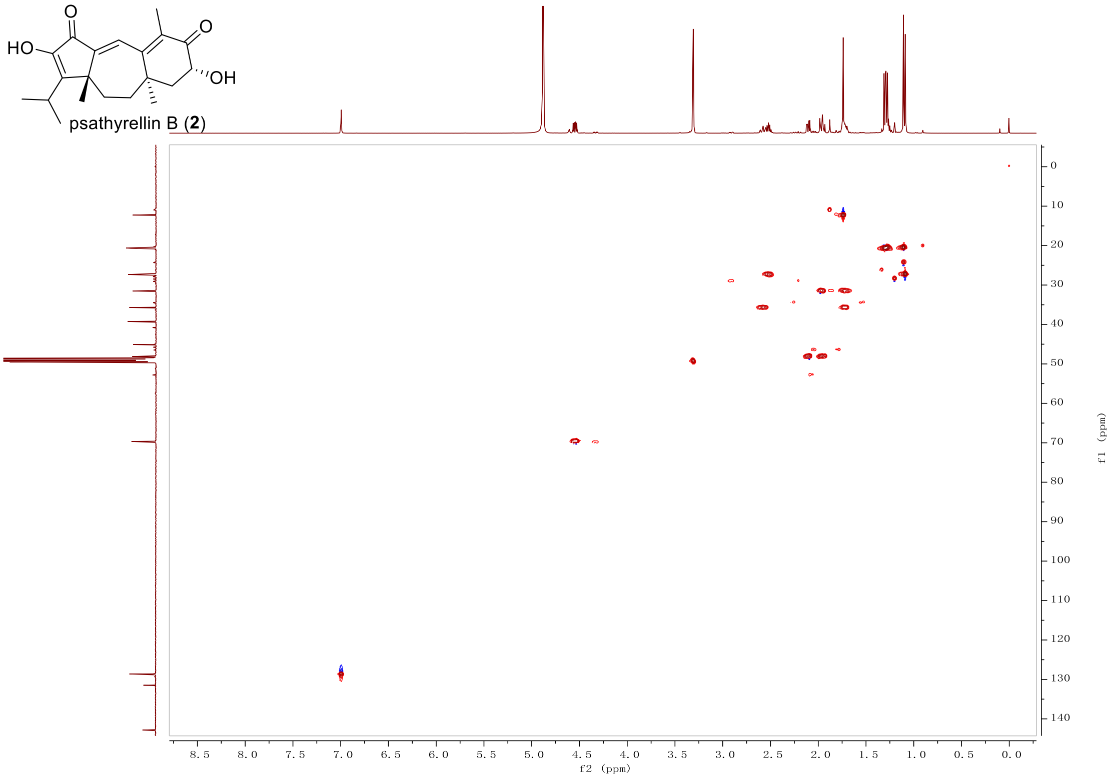

Figure 12S. HMBC spectrum of psathyrellin B (**2**)

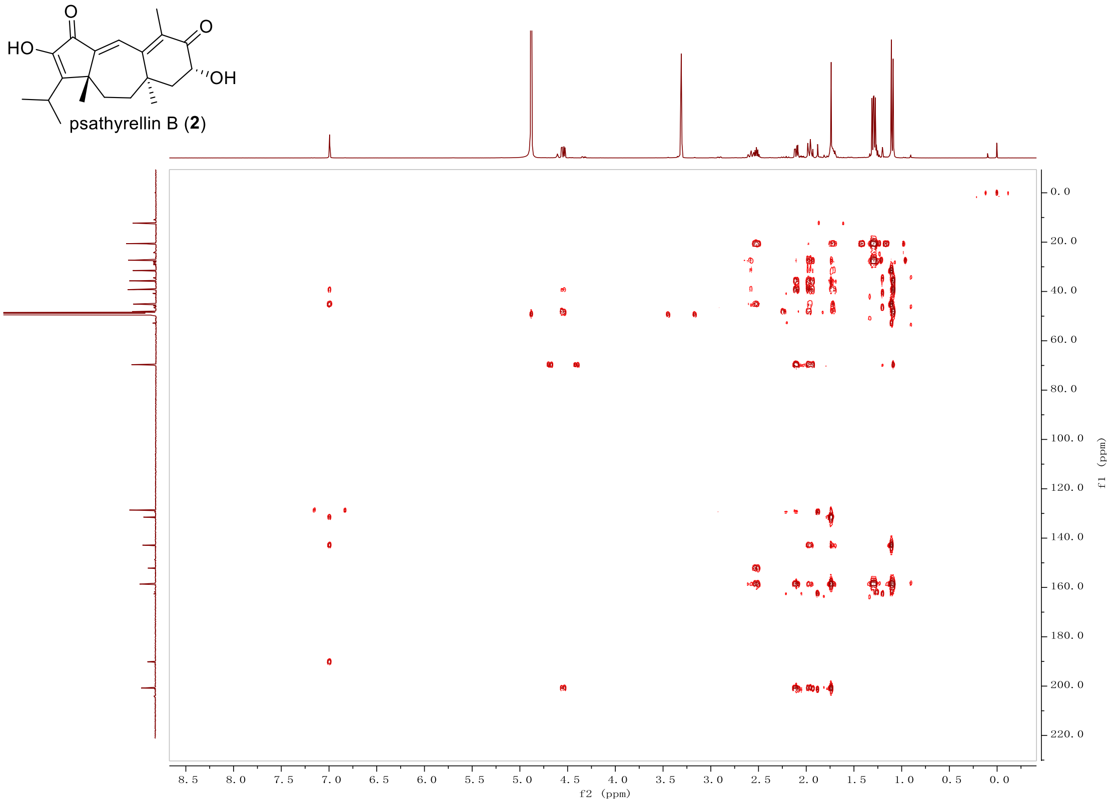

Figure 13S. ROESY spectrum of psathyrellin B (**2**)

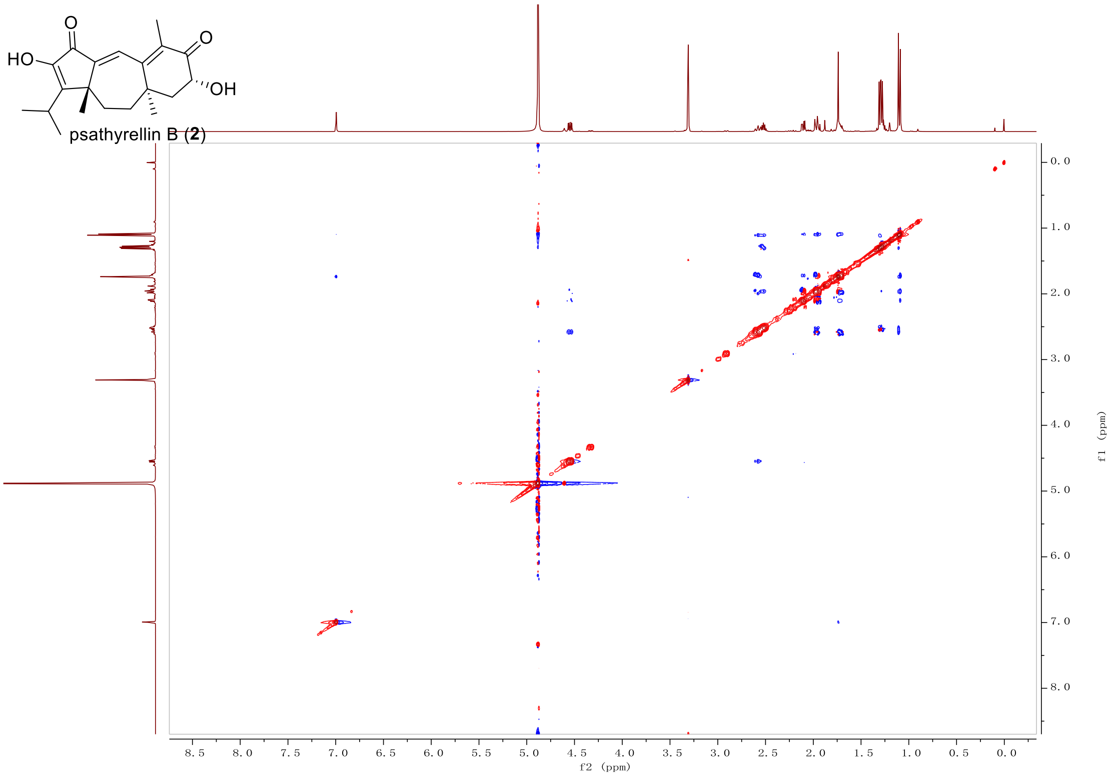

Figure 14S. HRESIMS of psathyrellin B (**2**)

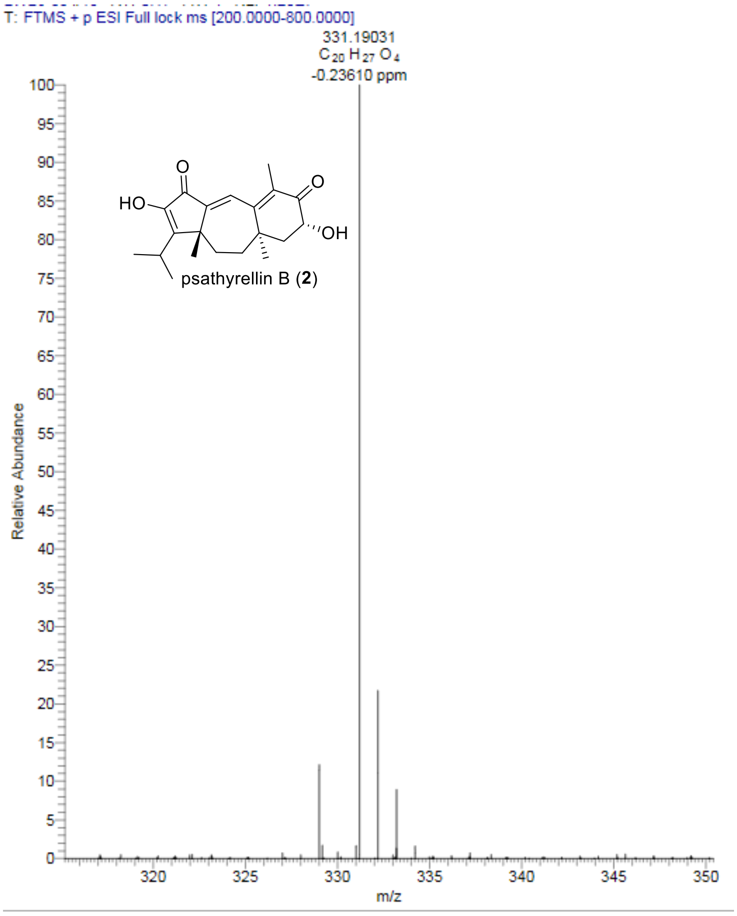

Figure 15S. CD spectrum of psathyrellin B (**2**)

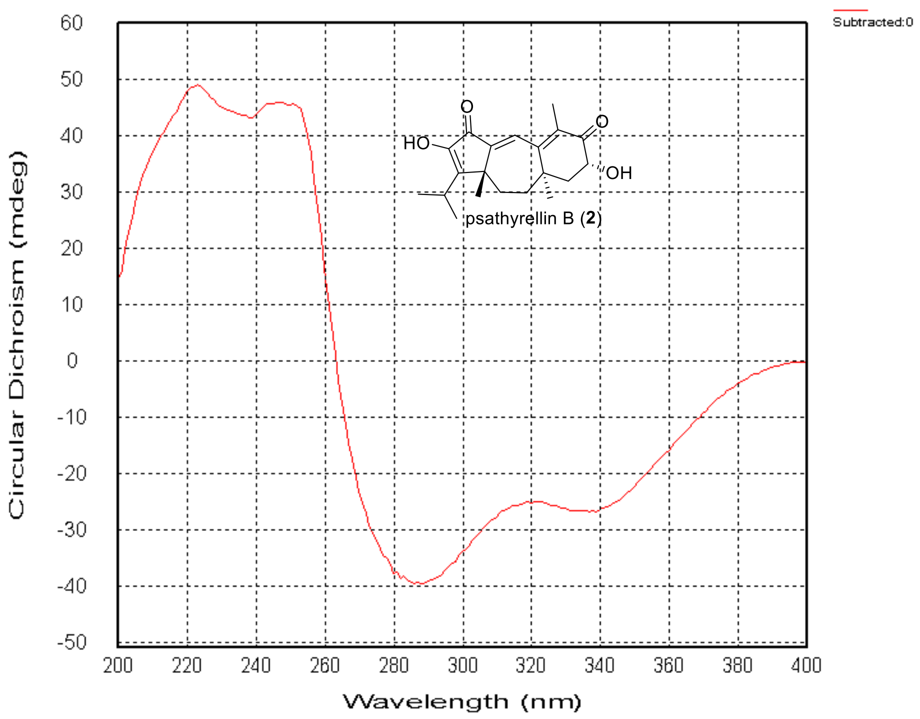

Figure 16S. ^{1}H NMR (600 MHz, methanol-d_4) spectrum of psathyrellin C (**3**)

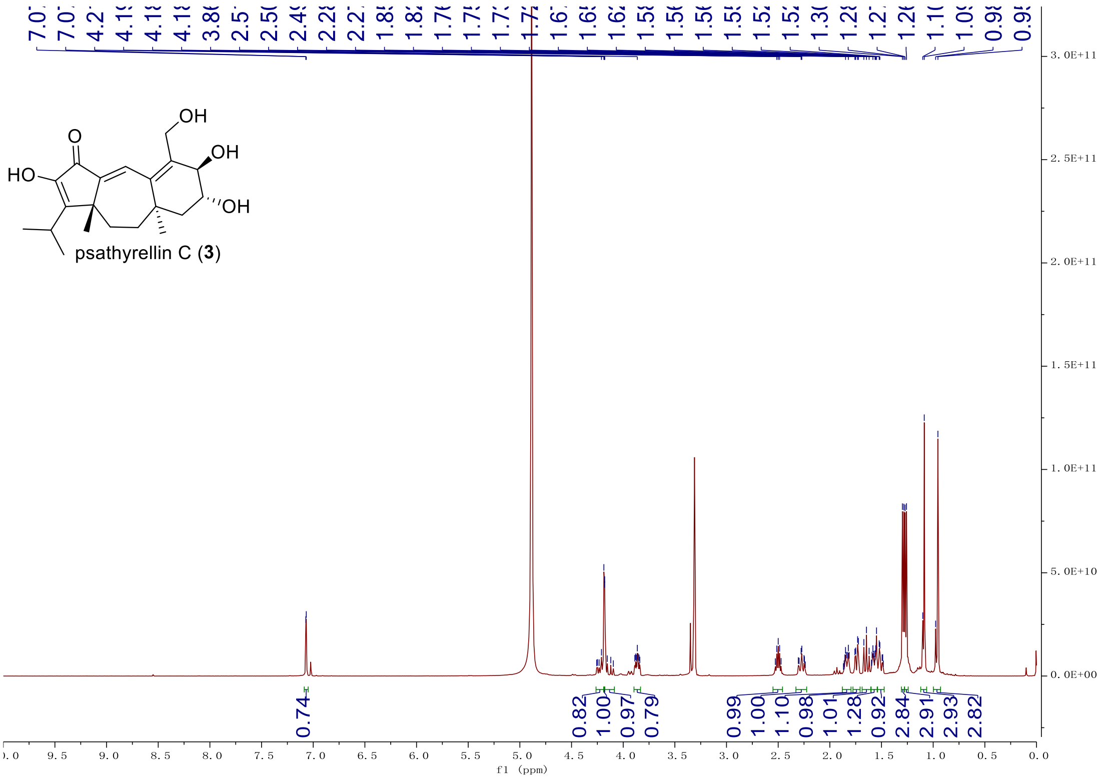

Figure 17S. ^{13}C NMR (150 MHz, methanol-d_4) spectrum of psathyrellin C (**3**)

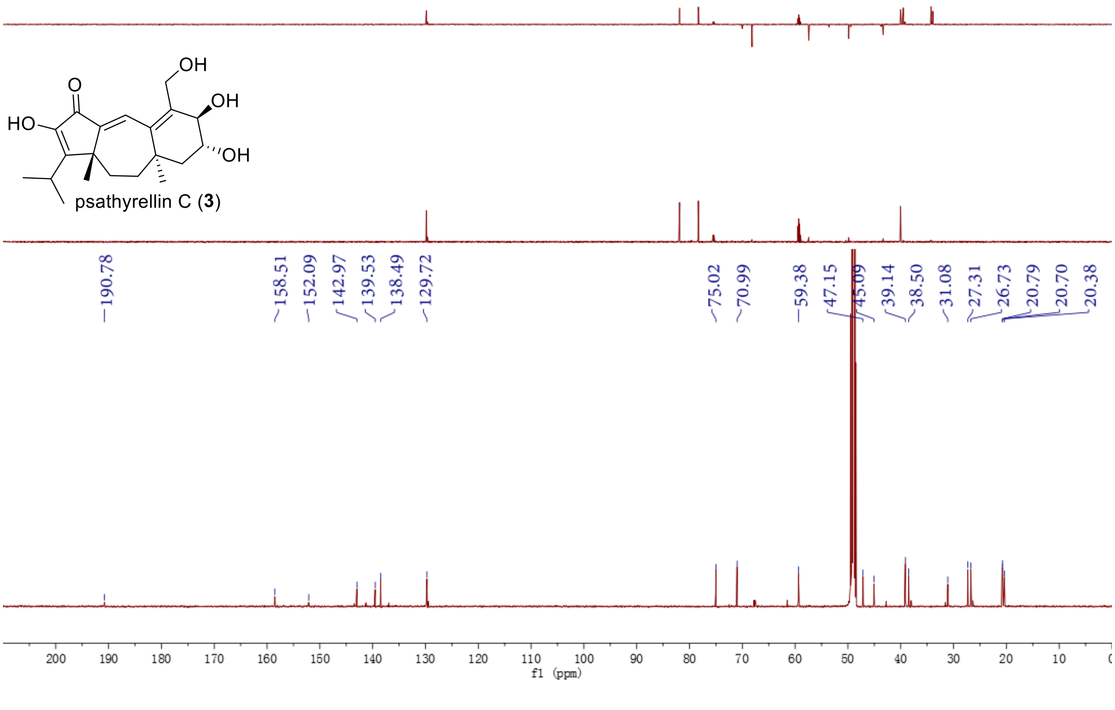

Figure 18S. ^{1}H-^{1}H COSY spectrum of psathyrellin C (**3**)

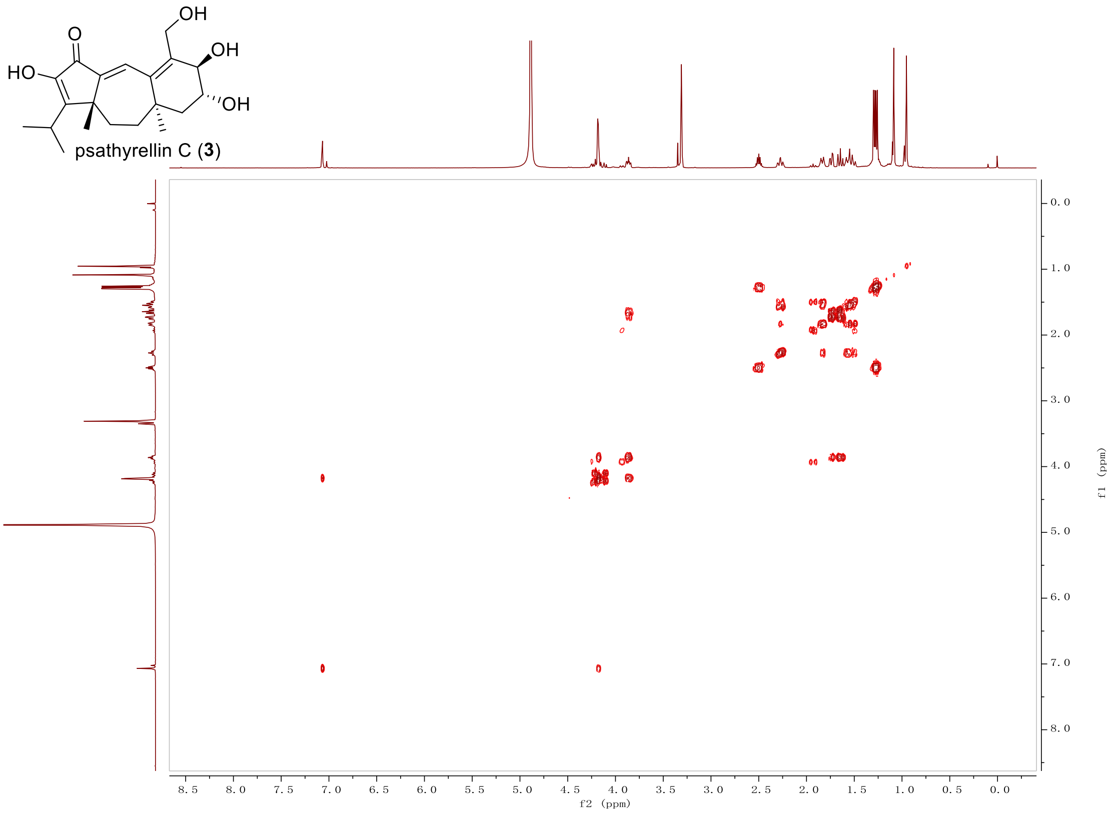

Figure 19S. HQSC spectrum of psathyrellin C (**3**)

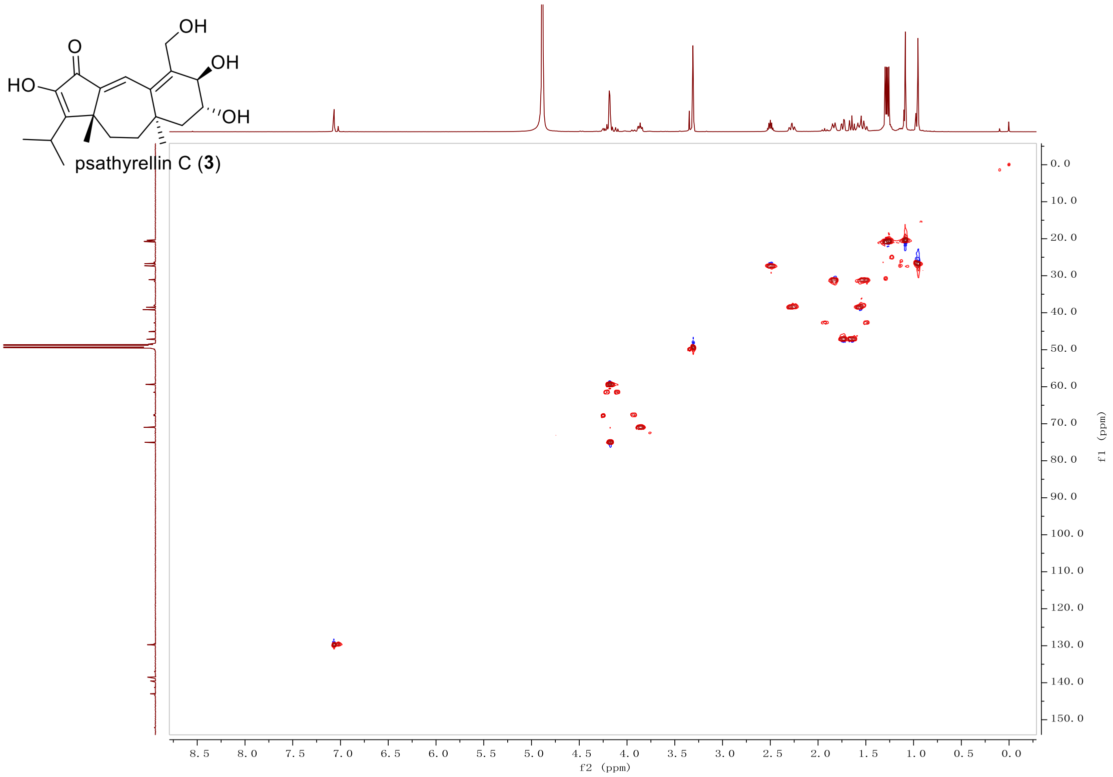

Figure 20S. HMBC spectrum of psathyrellin C (**3**)

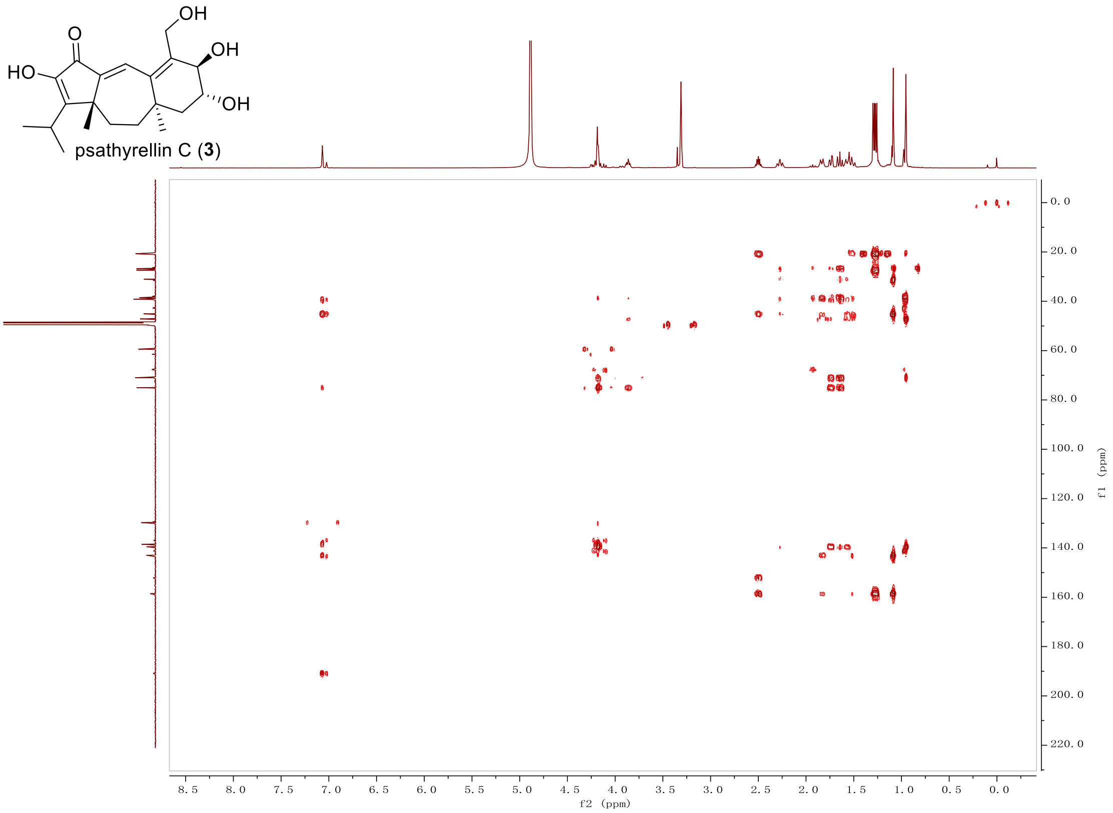

Figure 21S. ROESY spectrum of psathyrellin C (**3**)

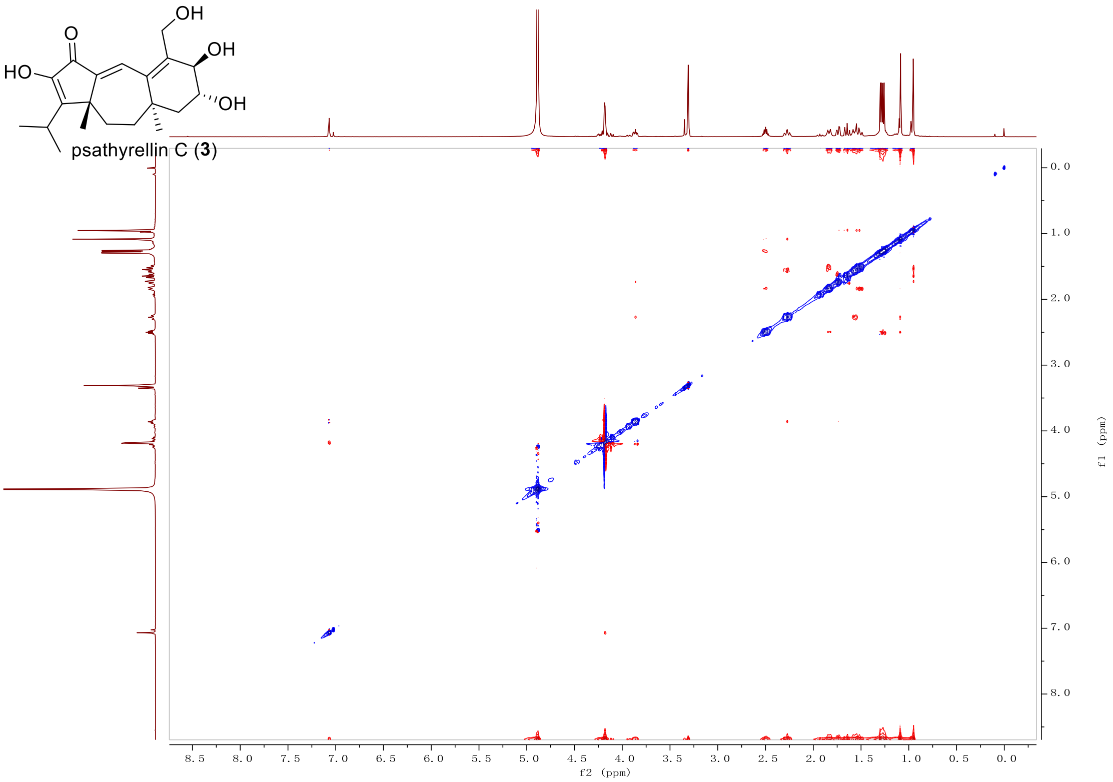

Figure 22S. HRESIMS of psathyrellin C (**3**)

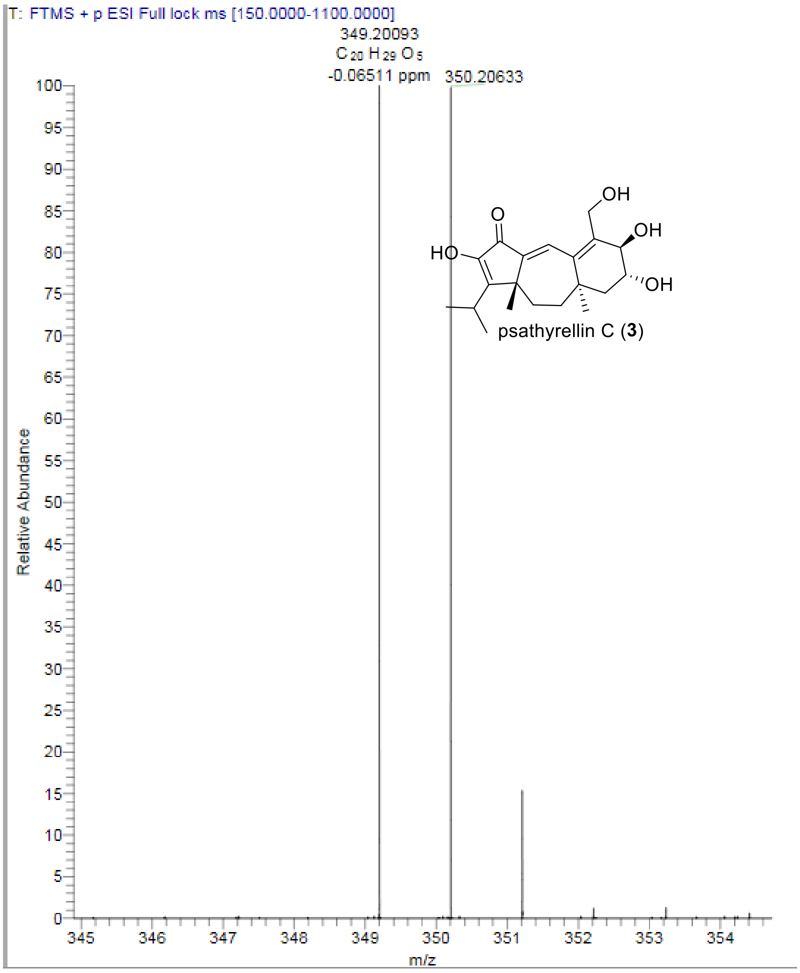

Figure 23S. CD spectrum of psathyrellin C (**3**)

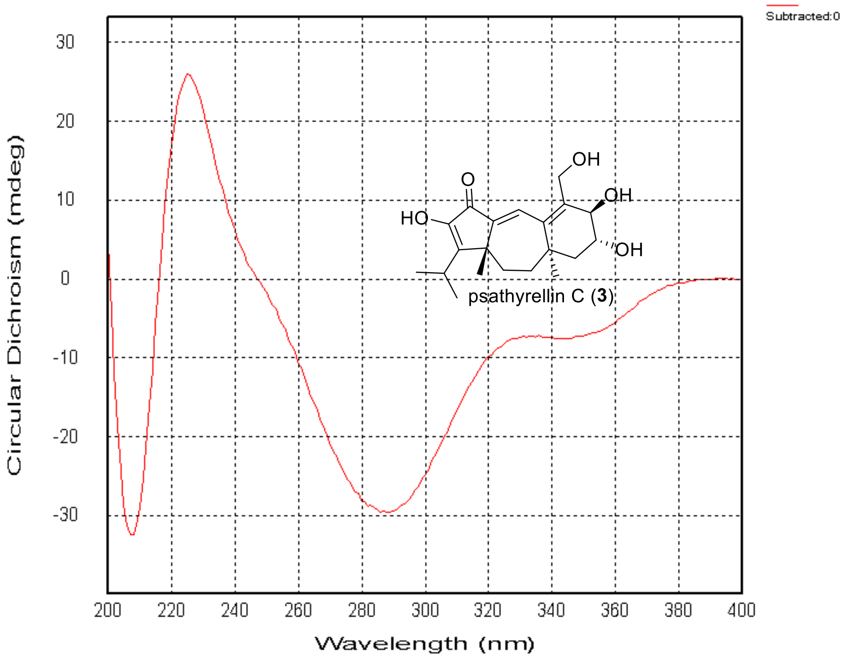

Figure 24S. ^{1}H NMR (600 MHz, methanol-d_4) spectrum of psathyrellin D (**4**)

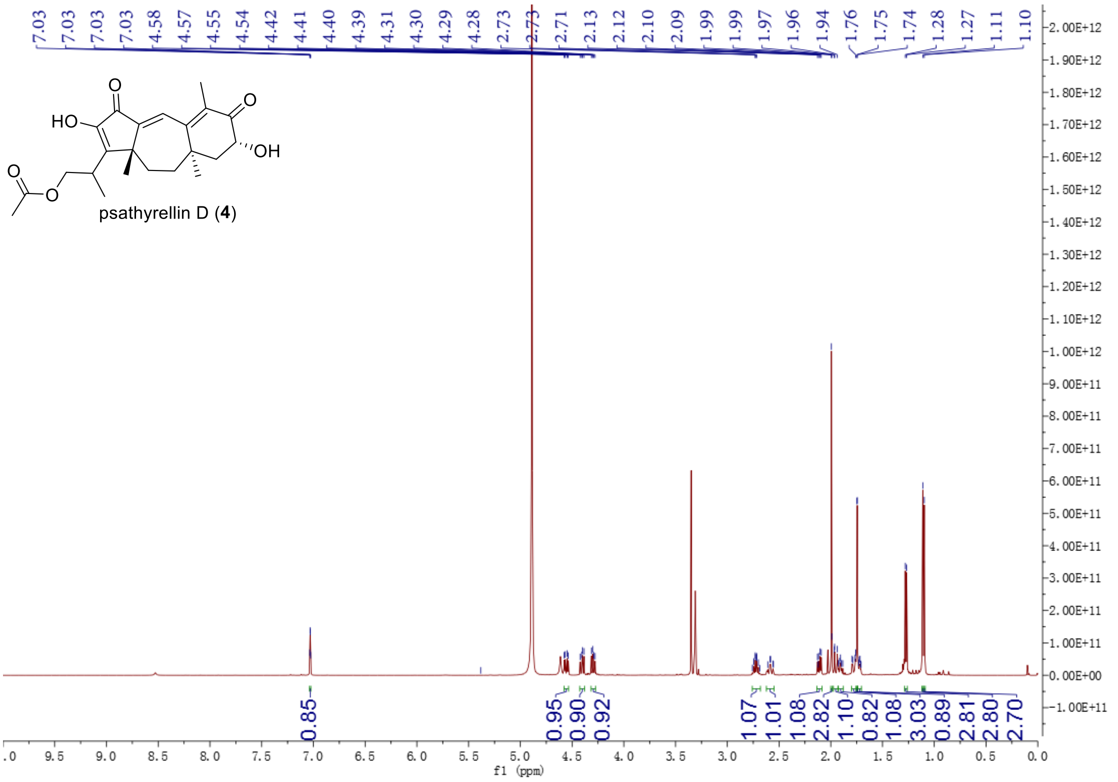

Figure 25S. ^{13}C NMR (150 MHz, methanol-d_4) spectrum of psathyrellin D (**4**)

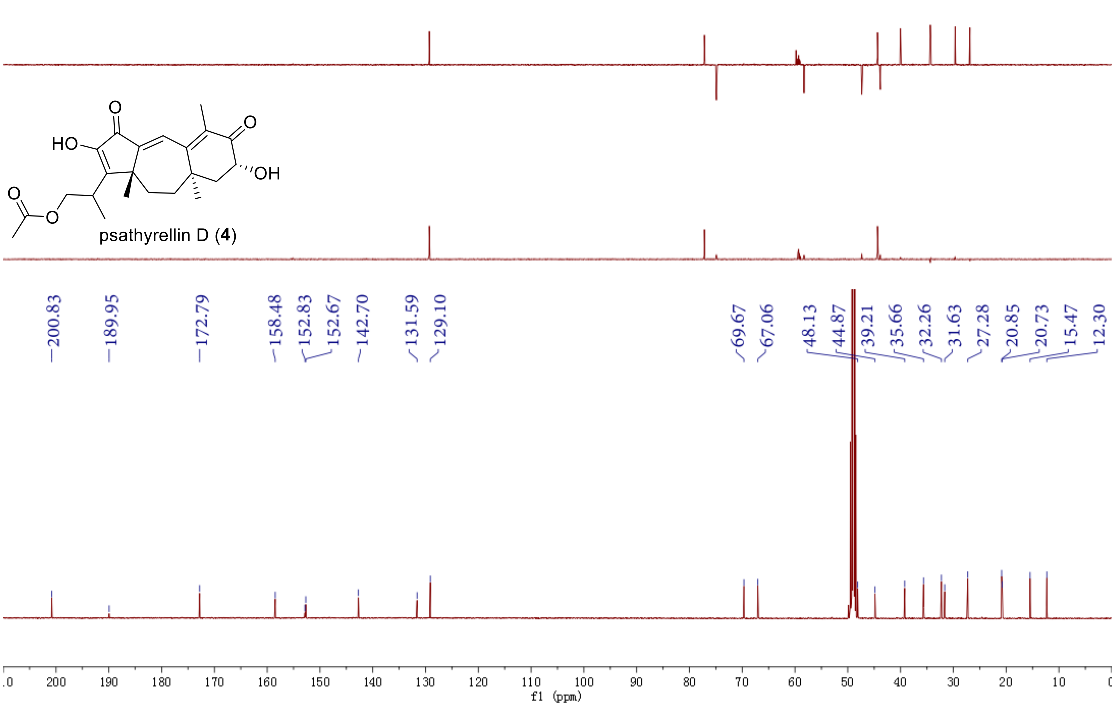

Figure 26S. ^{1}H-^{1}H COSY spectrum of psathyrellin D (**4**)

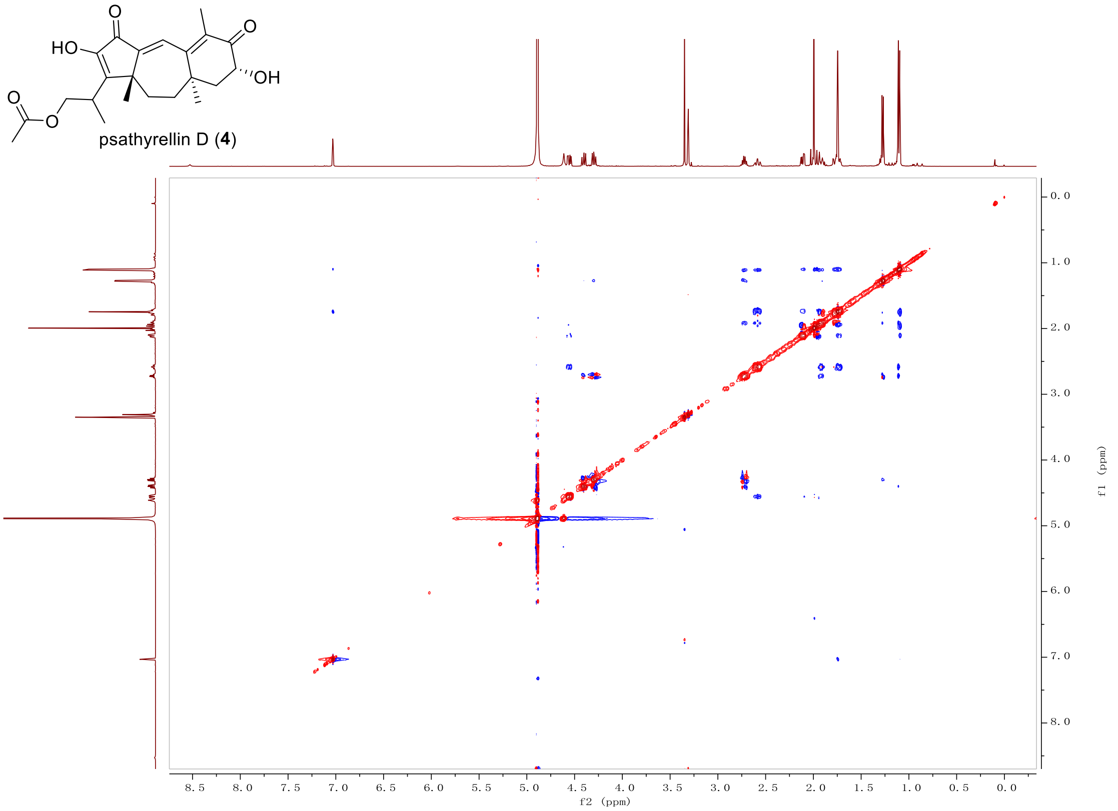

Figure 27S. HQSC spectrum of psathyrellin D (**4**)

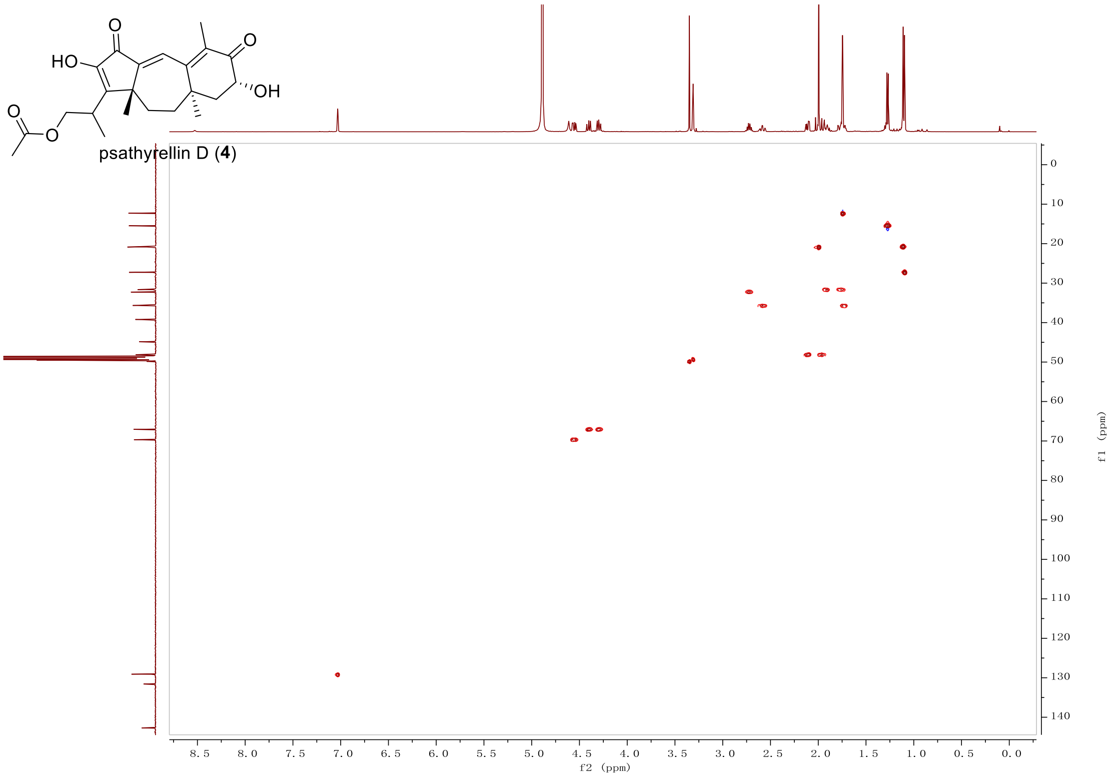

Figure 28S. HMBC spectrum of psathyrellin D (**4**)

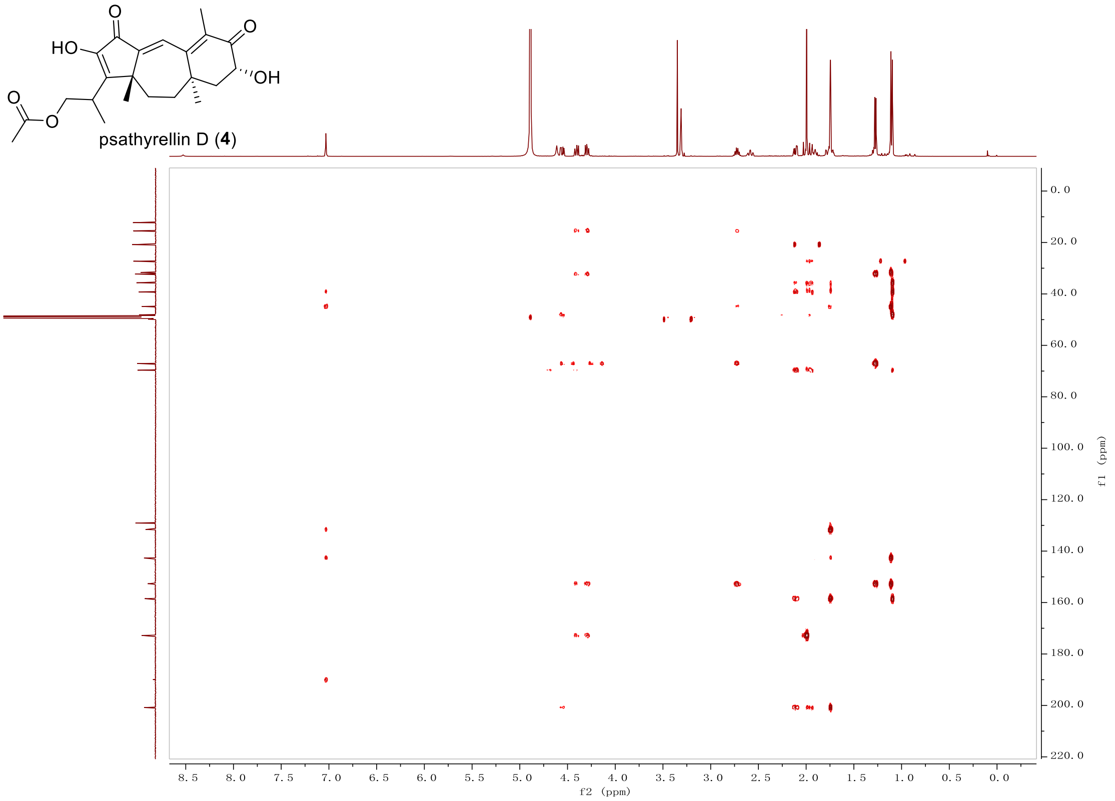

Figure 29S. ROESY spectrum of psathyrellin D (**4**)

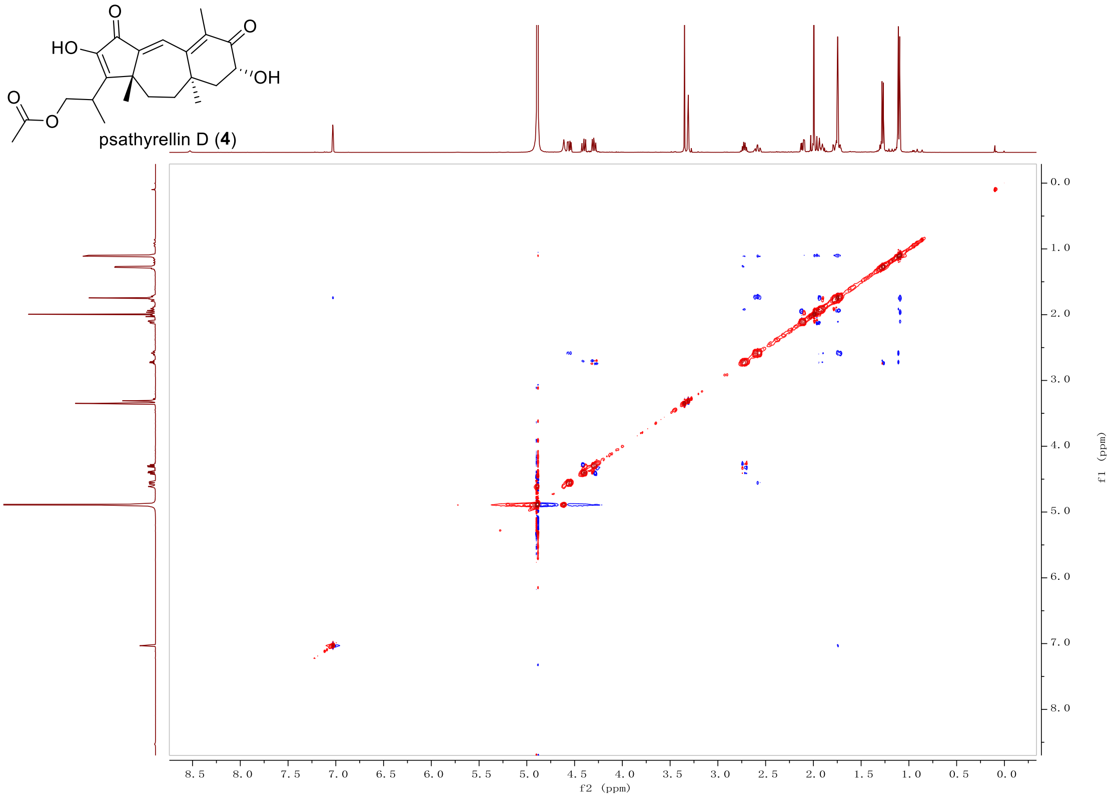

Figure 30S. HRESIMS of psathyrellin D (**4**)

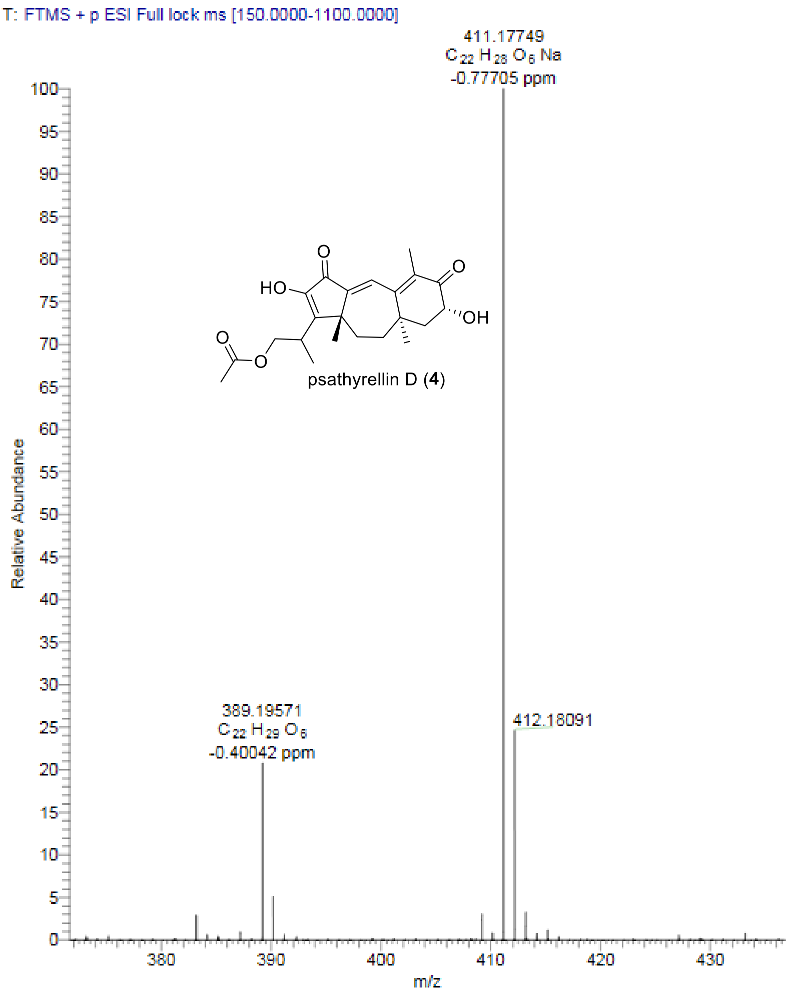

Figure 31S. CD spectrum of psathyrellin D (**4**)

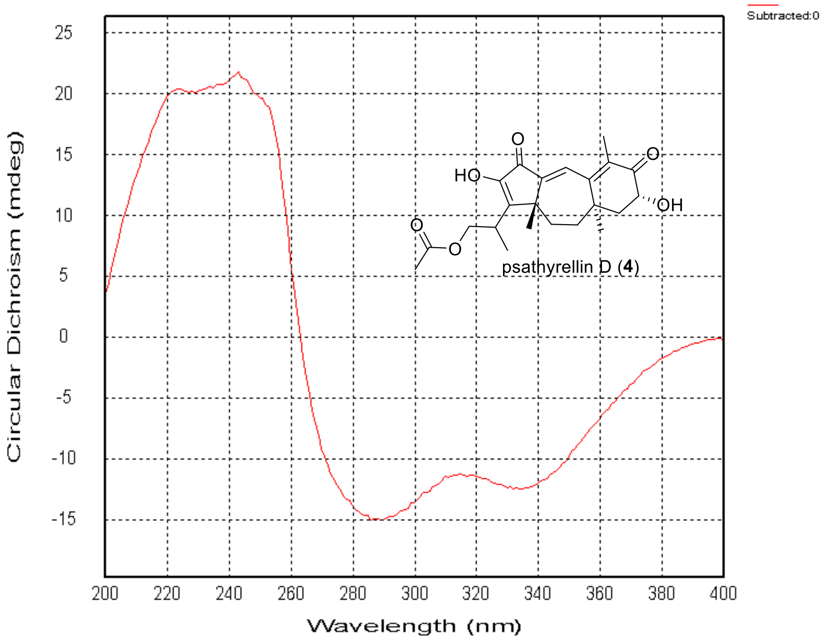

Figure 32S. ^{1}H NMR (600 MHz, methanol-d_4) spectrum of psathyrellin E (**5**)

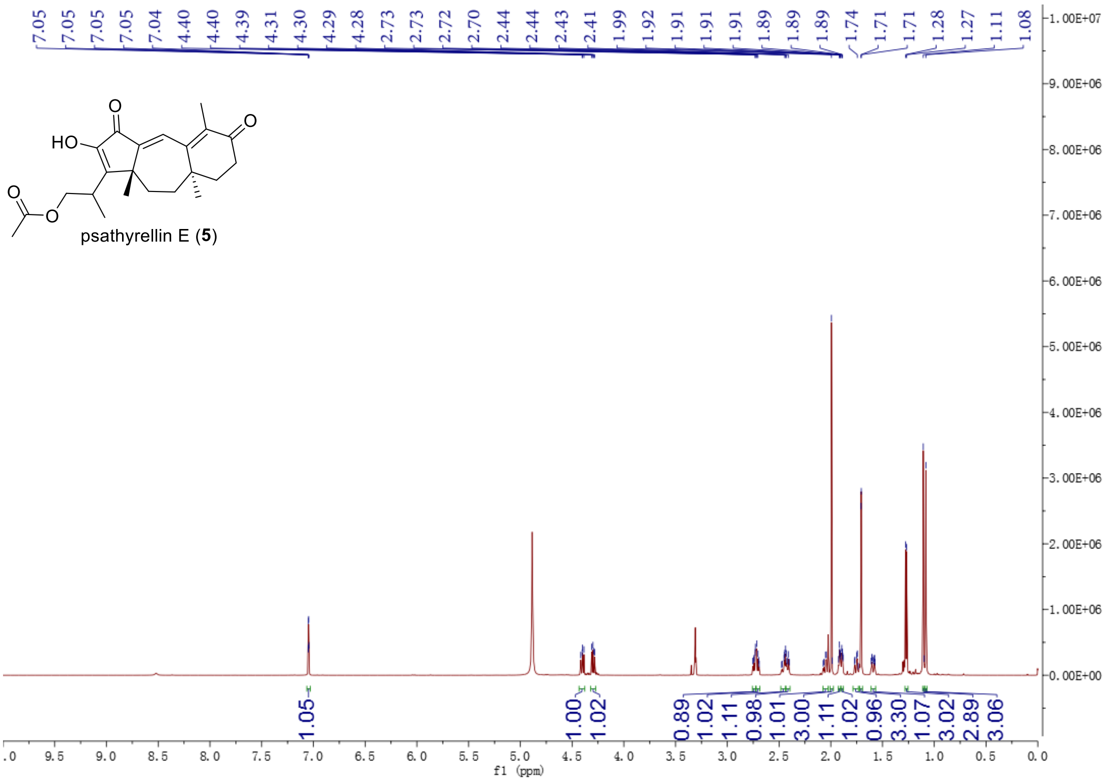

Figure 33S. ^{13}C NMR (150 MHz, methanol-d_4) spectrum of psathyrellin E (**5**)

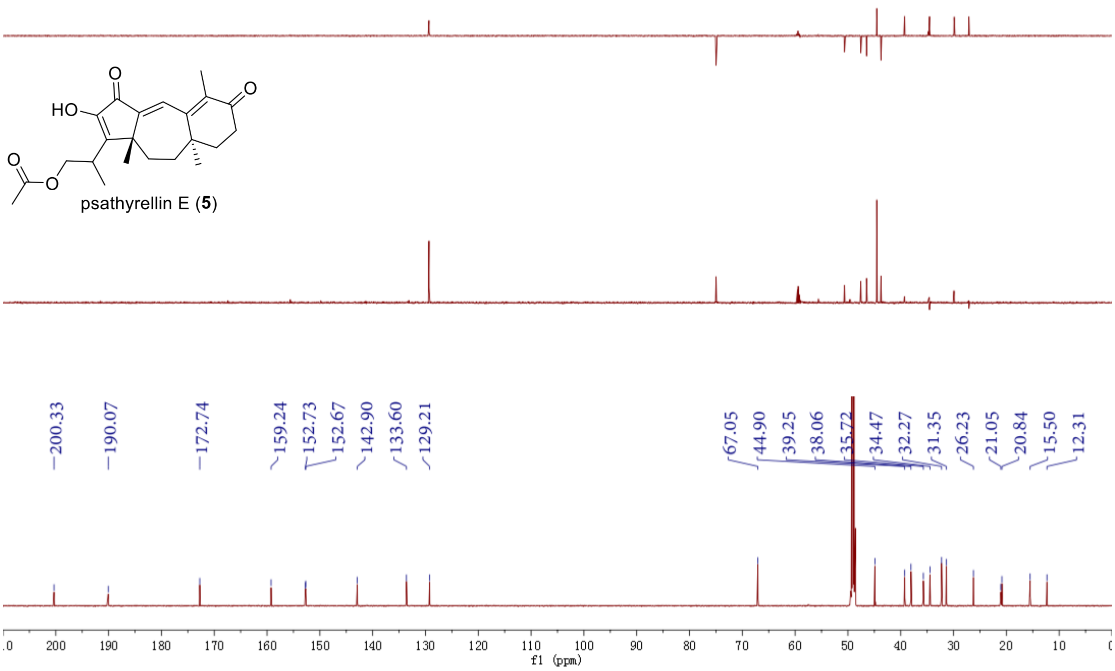

Figure 34S. ^{1}H-^{1}H COSY spectrum of psathyrellin E (**5**)

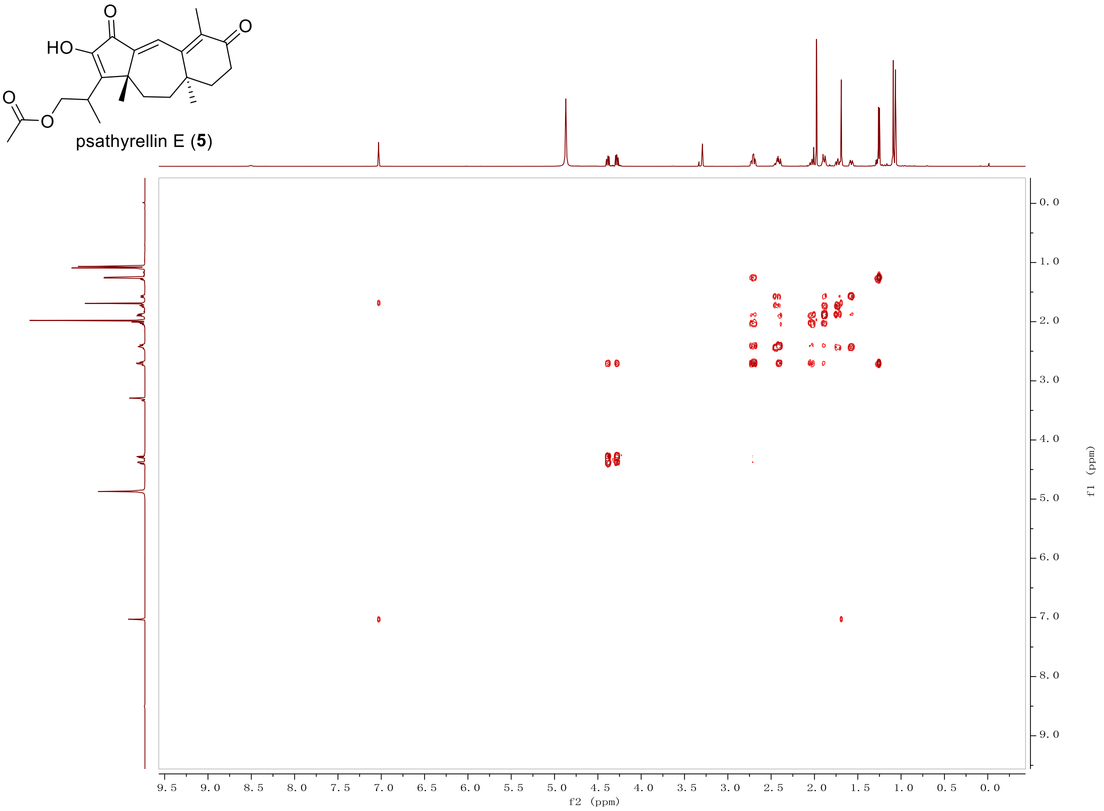

Figure 35S. HQSC spectrum of psathyrellin E (**5**)

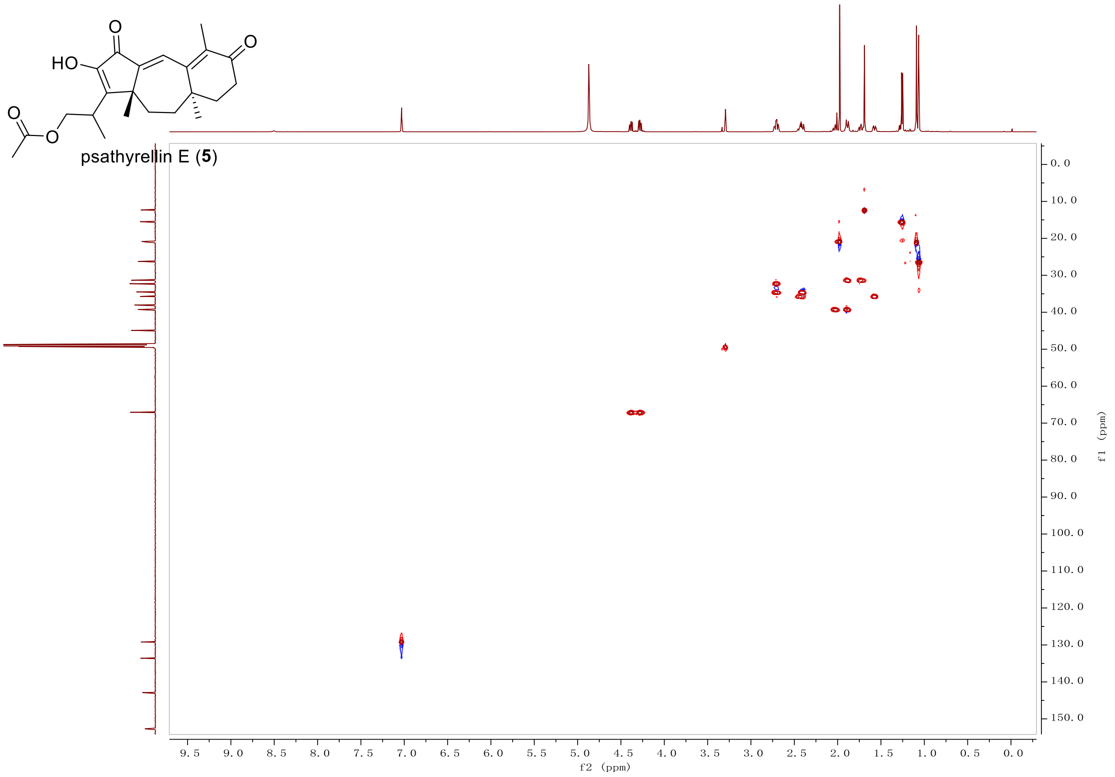

Figure 36S. HMBC spectrum of psathyrellin E (**5**)

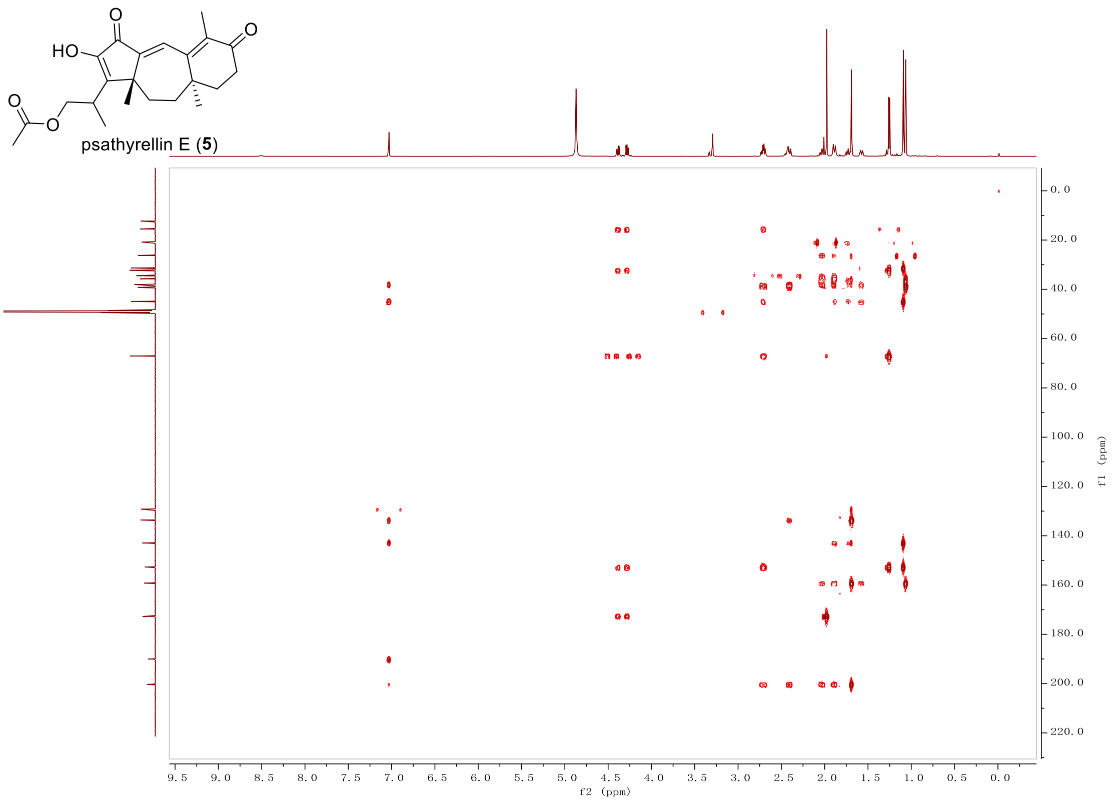

Figure 37S. ROESY spectrum of psathyrellin E (**5**)

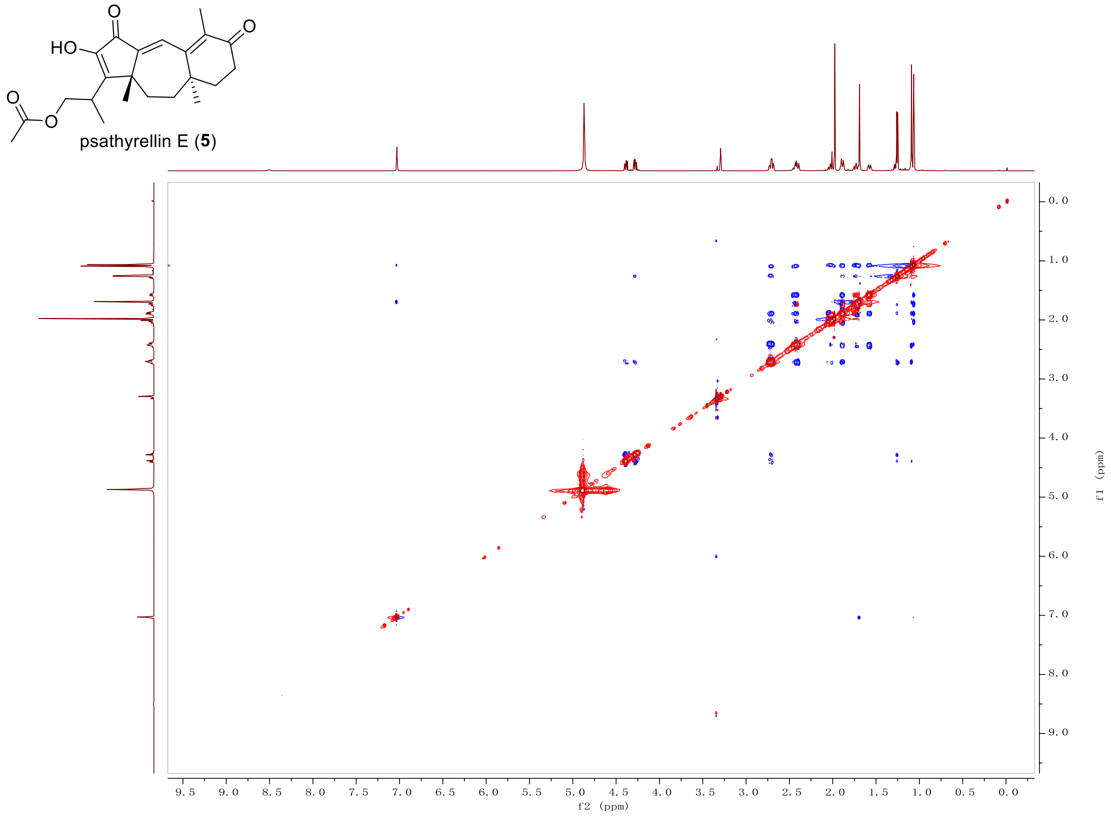

Figure 38S. HRESIMS of psathyrellin E (**5**)

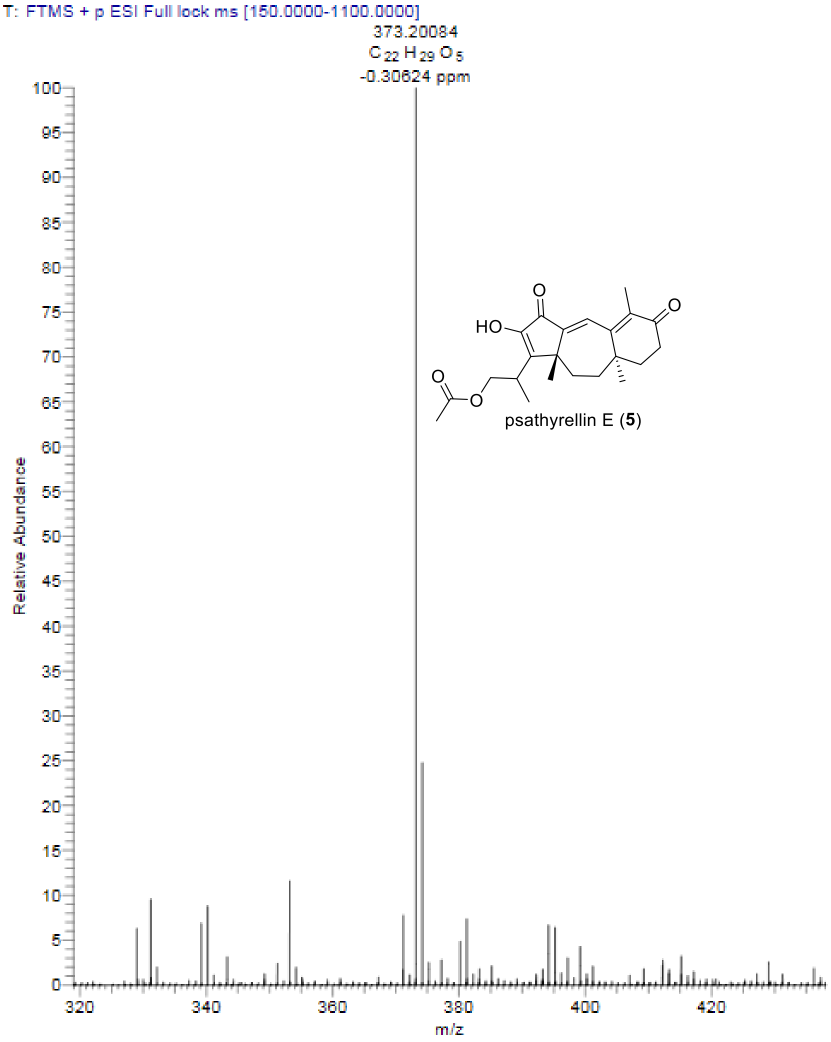

Figure 39S. CD spectrum of psathyrellin E (**5**).

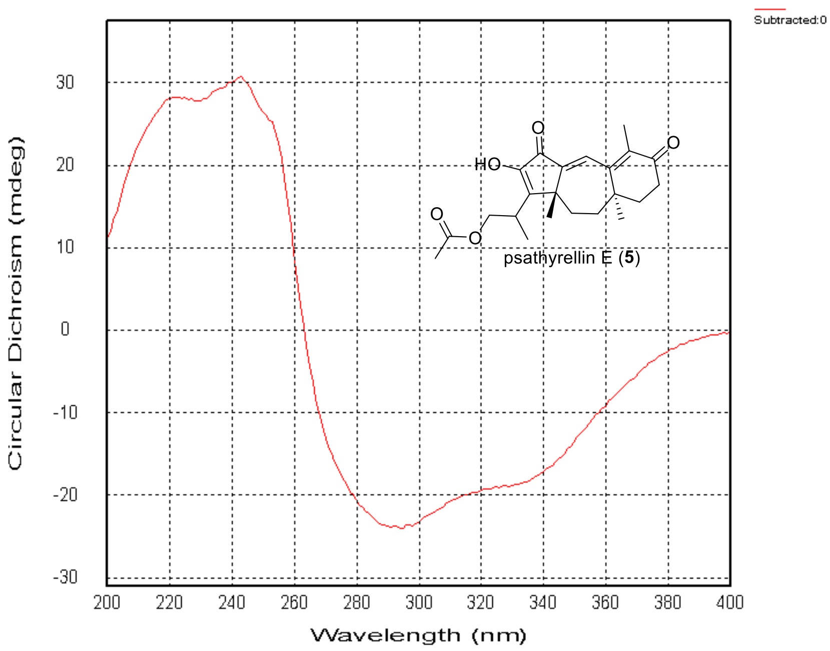

Supplement: Supplementary file 1 — Supplementary file1 (PDF 2820 KB) [file 13659_2021_316_MOESM1_ESM.pdf]
